# Pyrimidine-Mediated Electronic Modulation of Ionic Carbon Nitrides for Green Light-Driven Photocatalysis

**DOI:** 10.1021/acsmaterialsau.6c00080

**Published:** 2026-07-03

**Authors:** Anna Lo Presti, Serge Arnold Benedoue, Sonia Zoltowska, Sue-Faye Ng, Sergio Navalón, Christian Mark Pelicano

**Affiliations:** † Department of Colloid Chemistry, 28321 Max Planck Institute of Colloids and Interfaces, Potsdam 14476, Germany; ‡ Department of Inorganic Chemistry, Faculty of Science, 107751 University of Yaoundé 1, P.O. Box 812, Yaoundé 812, Cameroon; § Department of Chemistry, 16774 Universitat Politècnica de València, C/Camino de Vera, s/n, 46022 Valencia, Spain

**Keywords:** ionic carbon nitrides, poly(heptazine imides), green light-driven photocatalysis, heterogeneous photocatalysis, C–N cross coupling

## Abstract

Solar-driven organic transformations offer a sustainable strategy for the synthesis of high-value fine chemicals. However, most photoredox systems rely on high-energy light and costly molecular photosensitizers. Developing robust heterogeneous photocatalysts capable of efficient operation under low-energy visible light remains a critical challenge. Herein, we present a pyrimidine-mediated electronic modulation strategy to engineer ionic carbon nitrides for green-light-driven photocatalysis. Incorporation of 2,4,6-triaminopyrimidine during the thermal polymerization of potassium poly­(heptazine imide) induced controlled carbon doping within the framework, enabling band structure tuning, extended visible-light absorption, and enhanced charge separation and transport. The optimized material (0.5T-K) exhibited significantly improved photophysical properties, which translated into efficient catalytic performance under green-light irradiation. As a representative demonstration, the material enabled C–N cross-coupling with up to 99% conversion and sustained activity in extended reactions, while maintaining good recyclability. This work establishes a general strategy for tailoring the electronic structure of ionic carbon nitrides and highlights their potential as efficient, fully heterogeneous photocatalysts for low-energy, sustainable photoredox transformations.

## Graphical abstract

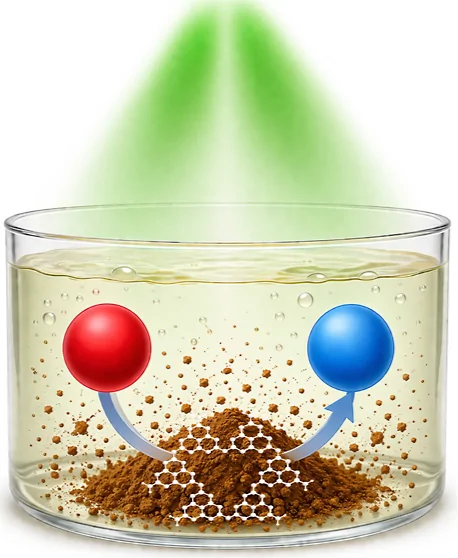

## 1 Introduction

The use of visible light as an energy input in photocatalysis has emerged as a powerful and sustainable strategy for chemical synthesis, enabling diverse transformations under mild conditions.^1,2^ Most photoredox systems rely on molecular photosensitizers to harvest light and initiate catalytic cycles.^3^ While homogeneous photocatalysts, particularly iridium- and ruthenium-based complexes, have demonstrated remarkable efficiency,^4^ their broader adoption is limited by high cost, scarcity of noble metals, and challenges associated with catalyst recovery and recycling.^5^ In this context, heterogeneous photocatalysts offer a more cost-effective, combining robustness, recyclability, and scalability.^6^ Despite significant advances in applications such as water splitting, H_2_ generation, and pollutant degradation, their use in bond-forming organic transformations remains comparatively underexplored.^7^ Among these transformations, C–N bond formation is of fundamental importance due to its prevalence in pharmaceuticals, agrochemicals, and functional materials.^8^ Photoredox/nickel dual catalysis has emerged as a versatile approach for C–N cross-coupling under mild conditions, building on the foundations of traditional palladium-catalyzed methodologies.^9–11^ However, most reported systems still depend on homogeneous photocatalysts and high-energy blue-light irradiation,^12,13^ limiting their sustainability and practical applicability. Developing heterogeneous photocatalysts capable of driving such transformations under lower-energy visible light therefore remains a significant challenge.

Metal poly­(heptazine imides) (*M*PHIs), a crystalline ionic carbon nitride allotrope, have emerged as highly promising metal-free polymeric semiconductors for photoredox catalysis.^14,15^ Their appeal stems from chemical stability, ease of processability, and tunable redox properties.^16,17^ These features make them versatile platforms for a wider range of applications, including water splitting, pollutant degradation, CO_2_ reduction,H_2_O_2_ generation, and various organic transformations.^18–22^ *M*PHIs have realized high apparent quantum yields (AQYs) under UV irradiation, enabled by precise control over by photophysical processes and associated reaction pathways. Building on these successes, a current challenge lies in broadening their light-harvesting capacity into the visible spectrum. This is an essential step toward maximizing solar energy utilization and advancing their viability in practical applications.^23,24^ Various strategies have been employed to achieve this goal, including heterojunction formation,^20,25^ heteroatom doping^26,27^ and nanostructuring,^22,28^ among others. Unlike heteroatom doping, which can introduce structural defects and promote charge recombination, uniform C doping offers distinct advantages. It enhances electron transport via the formation of extended delocalized π-systems and enables band structure tuning, broadening the solar coverage.^29^ Moreover, the uneven spatial distribution of C and N atoms induces surface charge asymmetry, which plays a vital role in boosting photocatalytic performance.^30^ In a pioneering study, Takanabe et al. utilized 2,4,6-triaminopyrimidine (TAP) to modulate the crystallinity of polytriazine imide, resulting in a notable enhancement of its AQY for sacrificial H_2_ evolution, achieving up to 15% at 400 nm.^31^ Another group reported a crystalline carbon nitride with electron-rich pyrimidine rings for H_2_ evolution couple with benzyl alcohol selective oxidation to benzaldehyde.^32^ While subsequent studies have primarily investigated carbon doping in covalent carbon nitrides,^33–35^ its effect on ionic carbon nitrides remains largely unexplored. Investigating pyrimidine-based carbon doping in these materials, particularly with respect to their photocatalytic activity under longer wavelengths, offers a new and promising direction.

In this work, we report a pyrimidine-mediated electronic modulation strategy to engineer ionic carbon nitrides with enhanced visible-light activity. Through the strategic incorporation of TAP during polymerization, carbon doping was successfully introduced into the PHI framework, enabling fine-tuning of its electronic structure. This modification not only enhanced visible-light absorption but also improved charge separation and mobility, key factors in driving efficient photocatalysis. To highlight the impact of this material design, the optimized catalyst (0.5T-K) enabled efficient green-light-driven C–N cross-coupling, achieving up to 99% conversion and sustained activity over extended reaction times. This work establishes a rational design strategy for tuning ionic carbon nitrides and highlights their potential as efficient, fully heterogeneous photocatalysts for low-energy, sustainable photoredox transformations.

## 2 Experimental Section

### 2.1 Synthesis of Potassium Poly­(Heptazine Imide)

Following the methodology from our previous publication, potassium poly­(heptazine imide) (KPHI) was synthesized. The process started by placing 2.5 g of dried 5-Amino-1H-tetrazole monohydrate and 12.5 g of a KCl/LiCl eutectic mixture (in a 0.55/0.45 ratio) into a steel ball mill vessel, which was then ground at a frequency of 25 Hz for 5 min. The resulting white powder was transferred into a lidded porcelain crucible and subjected to a heating process. The temperature in the furnace was gradually increased to 600 °C at a rate of 2.3 °C per minute under a continuous nitrogen flow (4 L/min), where it was held for 4 h. After the furnace was allowed to cool naturally to room temperature, the product was moved to a beaker with deionized water and stirred at room temperature overnight. Finally, the mixture was vacuum filtered, extensively washed with water via centrifugation, and dried overnight in a vacuum oven at 60 °C.

### 2.2 Synthesis of 2,4,6-Triaminopyrimidine-Modified KPHI Catalysts (xT-KPHI)

To obtain *xT-K* with varying TAP content, TAP was introduced at 0.5–5 wt % relative to the weight of 5-Amino-1H-tetrazole. The synthesis of *xT-K* catalysts followed a procedure similar to that of KPHI, with this minor modification. After this adjustment, the subsequent steps mirrored those used for KPHI synthesis. The final products were labeled as *xT-K*, where x represents 0.5, 1, 3, and 5, respectively.

### 2.3 Dual Nickel Photoredox C–N Cross-Coupling

In a screw-cap 5 mL vial equipped with a magnetic stir bar, 0.20 mmol (36.4 mg) of 4-bromobenzonitrile was dissolved in 1 mL of *N*,*N*-dimethylacetamide (DMA). Subsequently, 0.36 mmol (29.5 μL) of pyrrolidine, 0.44 mmol (49.3 mg) of DABCO, 5 mol % (3 mg) of NiBr_2_(dme), and 12 mg of the photocatalyst were added. The vial was then purged with argon for several minutes and tightly sealed. The reaction mixture was stirred under green LED light irradiation (λ = 530 nm) for 24 h. After completion, the suspension was centrifuged to remove the catalyst, and the supernatant was analyzed by GC-MS to quantify the product yield.

### 2.4 General Procedure for Catalyst Stability Tests

The reaction was performed in a 5 mL vial as previously described. In the first experiment, the recovered catalyst was washed with ethanol, dried, and then reused for another reaction. After 24 h of irradiation, the catalyst was recovered by centrifugation, washed with ethanol, and dried at 60 °C in a vacuum oven. After each catalytic run, the reaction mixture was collected and analyzed by GC-MS.

In the second experiment, the NiBr_2_(dme) was replenished. After the first catalytic run, the catalyst was recovered, washed with ethanol and dried at 60 °C. For the second reaction cycle, the recovered catalyst was combined with an additional NiBr_2_(dme) (3 mg, 5% mol) and the standard reaction mixture. After 24 h of irradiation, the catalyst was again recovered, washed, and dried. This procedure was repeated for a third cycle with the addition of a fresh portion of the NiBr_2_(dme) ((3 mg, 5% mol). After each catalytic run, the reaction mixture was collected and analyzed by GC-MS.

### 2.5 General Procedure for Scale-Up Experiment

In a three-neck reactor equipped with a magnetic stirring bar, 0.5T-KPHI (240 mg), NiBr_2_(dme) (60 mg), 4-bromobenzonitrie (728 mg, 10 mmol) and DABCO (986 g, 22 mmol) were placed and thoroughly mixed. Subsequently, DMA (20 mL), pyrrolidine (512 mg, 18 mmol), were added to the mixture. The reactor was then degassed with Ar, and the temperature sensor was immersed. The reaction mixture was irradiated for 24 h inside a green photoreactor. After the 24 h reaction period, the reaction mixture (1 mL) was extracted, the catalyst was separated by centrifugation, and the solution was analyzed by GC-MS. Reaction was carried out in total for 96 h.

### 2.6 General Procedure for Reactions with Different Aryl Halides and Amines

A 5 mL screw-cap vial equipped with a magnetic stir bar was charged with 0.5T-KPHI (12 mg), NiBr_2_(dme) (5 mol %, 3 mg), and DABCO (49.3 mg, 0.44 mmol). Subsequently, DMA (1 mL), the selected aryl halide (0.20 mmol), and pyrrolidine (0.36 mmol) were added. For experiments involving different amines, 4-bromobenzonitrile (0.20 mmol) and the corresponding amine (0.36 mmol) were used instead. The vial was sealed with a screw cap fitted with a PTFE liner and degassed with argon using a double-needle technique. The reaction mixture was then irradiated under green LED light (λ = 530 nm) for 24 h in a photoreactor. After irradiation, the solid catalyst was removed by centrifugation, and the supernatant was transferred back to the vial for GC-MS analysis.

### 2.7 Material Characterizations

X-ray powder diffraction (XRD) patterns were recorded using a Rigaku Smart Lab diffractometer (Japan) with Cu Kα radiation (λ = 0.154 nm), operated at 40 kV and 50 mA. Scans were performed from 5° to 80° (2θ) at a rate of 2°/min. X-ray photoelectron spectroscopy (XPS) was conducted on a Thermo Fisher ESCALAB 250Xi system. Fourier-transform infrared (FTIR) spectra were collected using a Thermo Scientific Nicolet iD5 spectrometer equipped with an ATR accessory.

Thermogravimetric analysis (TGA) was performed on a Shimadzu TGA-60H instrument from 25 to 900 °C under synthetic air flow (50 mL/min) at a heating rate of 10 °C/min, using alumina crucibles. Inductively coupled plasma optical emission spectrometry (ICP-OES) was carried out with a PerkinElmer Optima 8000. Elemental analysis (CHNOS) was performed using a vario MICRO cube analyzer (Elementar Analysensysteme GmbH).

Nitrogen adsorption–desorption isotherms and pore size distributions were obtained at 77 K using a Quantachrome Quadrasorb SI analyzer. Prior to measurement, samples were degassed at 150 °C under 0.5 Torr vacuum for 15 h. Surface areas were calculated from the adsorption branch (P/P_0_ < 0.3) using the Brunauer–Emmett–Teller (BET) method.

Scanning electron microscopy (SEM) images were acquired on a Zeiss LEO 1550-Gemini microscope equipped with an Oxford Instruments X-MAX EDX detector. Transmission electron microscopy (TEM) was carried out on JEOL JEM-F200 and JEM-ARM 200F microscopes (with double Cs correction) operating at 80 kV, featuring a cold field-emission source and a high-angle silicon drift EDX detector (Jeol JED 2300) with a detection area of 100 mm^2^ and a solid angle up to 0.98 sr.

Optical properties and charge carrier behaviors were analyzed using UV–vis diffuse reflectance spectroscopy (UV–vis DRS, Shimadzu UV-2600), electron paramagnetic resonance (EPR, Bruker EMXnano), and steady-state photoluminescence (PL) spectroscopy (Jasco FP-8300) with excitation at 365 nm. Time-resolved photoluminescence (TRPL) measurements were obtained using a FluoTime 250 spectrometer (PicoQuant) equipped with a PDL 800-D picosecond pulsed diode laser. Average lifetimes (τ_ave_) were calculated using appropriate multiexponential fitting: (τ_ave_) = (A_1_ τ_1_^2^ + A_2_ τ_2_^2^ + A_3_ τ_3_^2^)/(A_1_ τ_1_ + A_2_ τ_2_ + A_3_ τ_3_).

### 2.8 Photoelectrochemical Measurements

Photoelectrochemical experiments were carried out in a conventional three-electrode configuration, employing a platinum wire as the counter electrode and an Ag/AgCl electrode as the reference. All measurements were conducted using a Gamry Interface 1010E potentiostat. To fabricate the working electrode, fluorine-doped tin oxide (FTO) glass slides (3 × 1 cm) were thoroughly cleaned by sequential sonication in detergent, deionized water, and ethanol for 15 min each. The catalyst ink was prepared by dispersing 5 mg of the photocatalyst in 0.5 mL of water containing 20 μL of 5 wt % Nafion solution, followed by 30 min of sonication. A 50 μL aliquot of the resulting suspension was drop-cast onto the cleaned FTO substrate, dried at 60 °C, and annealed at 120 °C for 1 h to improve film adhesion. All measured potentials were referenced to the reversible hydrogen electrode (RHE) using the following conversion: *E*_*RHE*_ = *E*_*Ag/AgCl*_ + 0.059 × pH + 0.197.

### 2.9 Transient Photocurrent Response

Photocurrent responses were recorded under white LED illumination (100 mW cm^–2^) in 0.5 M Na_2_SO_4_ aqueous electrolyte at an applied potential of 0 V vs Ag/AgCl using the same potentiostat.

### 2.10 Electrochemical Impedance Spectroscopy

EIS measurements were conducted using the same electrode setup over a frequency range from 10 kHz to 1 Hz. The impedance spectra were analyzed and fitted using Z-View software, typically showing a single semicircle.

### 2.11 Mott–Schottky Measurements

Mott–Schottky plots were obtained using a BioLogic MPG-2 workstation at multiple frequencies, employing the same three-electrode configuration described above.

## 3 Results and Discussion

### 3.1 Morphological and Structural Characterizations

The schematic representation of the carbon doping strategy for *K*PHI is depicted in Figures 1a and S1. Initially, a specified amount of 2,4,6-triaminopyrimidine (TAP) is combined with 5-aminotetrazole and a salt mixture of KCl/LiCl in a 0.55/0.45 mass ratio. This blend is then placed in a ball mill and ground at a frequency of 25 Hz for 5 min. The resultant powder is transferred to a crucible, covered, and subjected to annealing at 600 °C for 4 h in a N_2_ atmosphere. The resultant yellowish-brown powders are designated as *xT*-*K*PHI, where x represents the weight percentage of TAP relative to 5-aminotetrazole. For the synthesis of pure *K*PHI, the same process is followed but TAP is omitted. The evident doping effect is reflected in the color variation of the samples. While pristine *K*PHI exhibits a yellow hue, the samples progressively shift to orange and eventually brown as the TAP concentration increases from 0.5 to 5 wt %.

**Figure 1:**
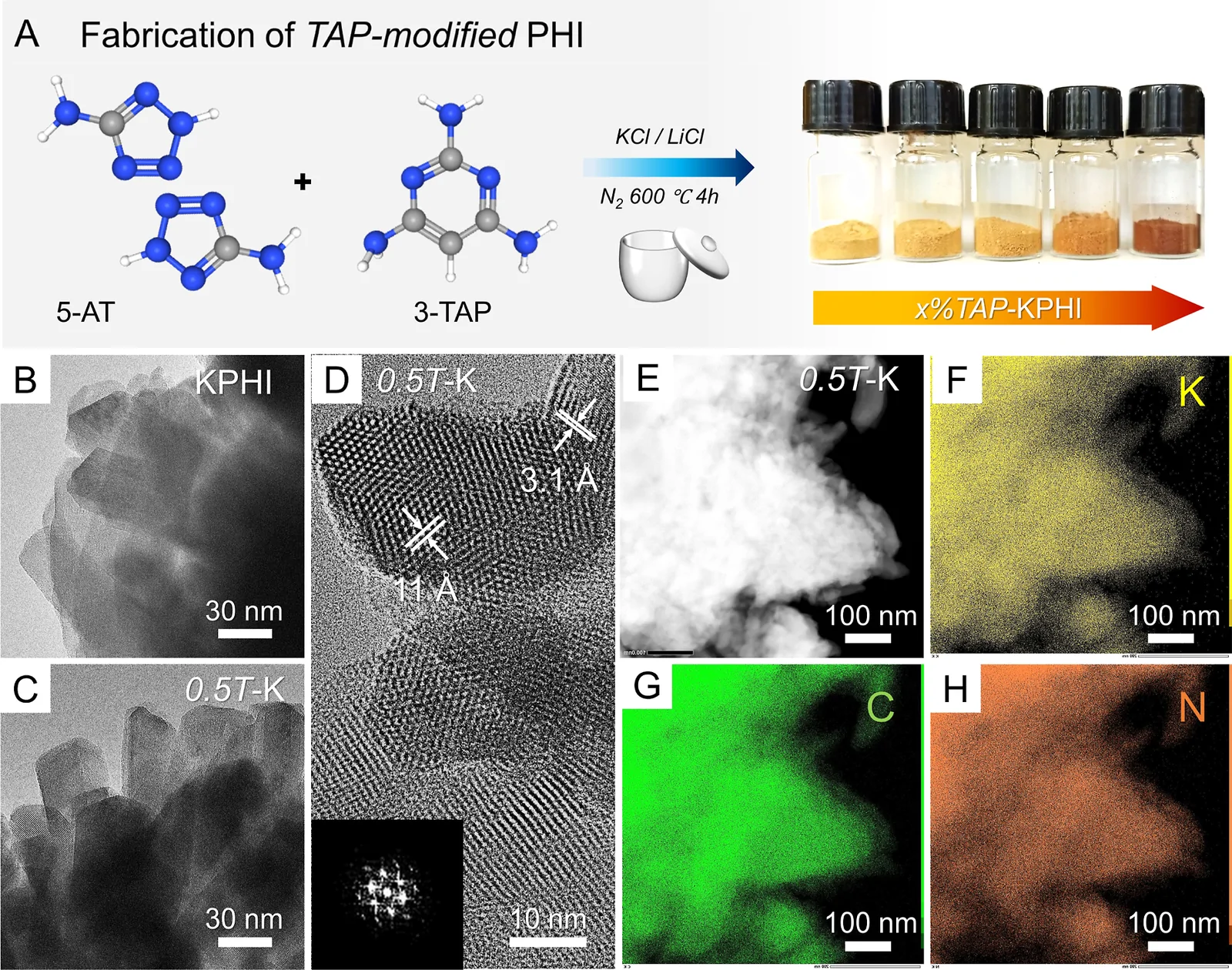
(A) Schematic diagram for preparing pyrimidine-modified KPHI. TEM images of (B) KPHI and (C) 0.5T-KPHI. (D) HRTEM image of 0.5T-KPHI (inset FFT). (E) High angle annular dark-field (HAADF)-STEM image of 0.5T-KPHI. (F–H) STEM-EDX elemental mapping images of (E).

The morphological evolution of *xT*-KPHI catalysts with increasing TAP content was studied using scanning electron (SEM) and transmission electron microscopy (TEM). As shown in Figures S2a and 1b, pristine *K*PHI unveils a nanorod structure with an average length of 200 nm. The *xT*-*K*PHI catalysts preserved this architecture (Figures S2 and 1c), except in the case of the 5T-K sample, where smaller crystallites appeared. This structural alteration suggests that TAP affected the polymerization process of *K*PHI. A closer examination of TEM images reveals that the introduction of 1 wt % TAP induced noticeable distortions in the rod-like crystals (Figure S3c). Further increasing the TAP content to 3 and 5 wt % resulted in the breakup of larger crystals, leading to their subsequent agglomeration (Figure S3d,e). A representative TAP-modified *K*PHI (0.5T-K) sample shows characteristic lattice fringes of 11 Å and 3.1 Å, which are ascribed to the (110) and (001) crystal planes, respectively (Figure 1d). Additionally, scanning TEM-energy dispersive spectroscopy (EDS) elemental mapping images validate the uniform distribution of K, C, and N elements within the catalyst area (Figure 1e–h).

To gain a deeper understanding of the chemical structure of the pyrimidine-modified KPHI framework, X-ray diffraction (XRD) and Fourier transform infrared (FTIR) spectroscopy were carried out. As shown in Figure 2a, all the samples exhibit two intense diffraction peaks at 2θ = 8.2° and 28.4°, corresponding to the in-plane structural packing (100) and interlayer aromatic stacking of heptazine motif (002), respectively.^36^ As the amount of TAP loading increases, the (002) peak progressively shifts toward lower angles, indicating an expanded interlayer spacing. The pronounced peak broadening and weaker diffraction signals suggest a higher defect density and a decline in crystallinity as the TAP concentration increases. This finding accounts for the partial distortion observed in the crystalline framework of *K*PHI following the doping process. Thermogravimetric analysis reveals that TAP functions not only as a dopant precursor but also influences the polymerization dynamics, owing to the gas-phase species released during its thermal decomposition (Figure S4). The FTIR spectra of *xT*-*K*PHI catalysts possess identical fingerprint signals to those of pure KPHI, confirming that TAP modification does not alter the PHI polymeric backbone (Figure 2b). The sharp absorption peak at 801 cm^–1^ corresponds to the out-of-plane bending mode of heptazine ring, while the absorption bands spanning 1200–1650 cm^–1^ range are attributed to the stretching vibrations of the heptazine derivatives.^37^ The distinct bands at 987 and 1065 cm^–1^ are ascribed to the symmetric and asymmetric vibrations of NC_2_ bonds in K–NC_2_ units. The presence of cyano moieties is confirmed by the absorption band at 2180 cm^–1^, whereas the broad bands observed between 3000 and 3700 cm^–1^ are indicative of residual NH_*x*_ groups and physisorbed water molecules on the catalyst surface.

**Figure 2:**
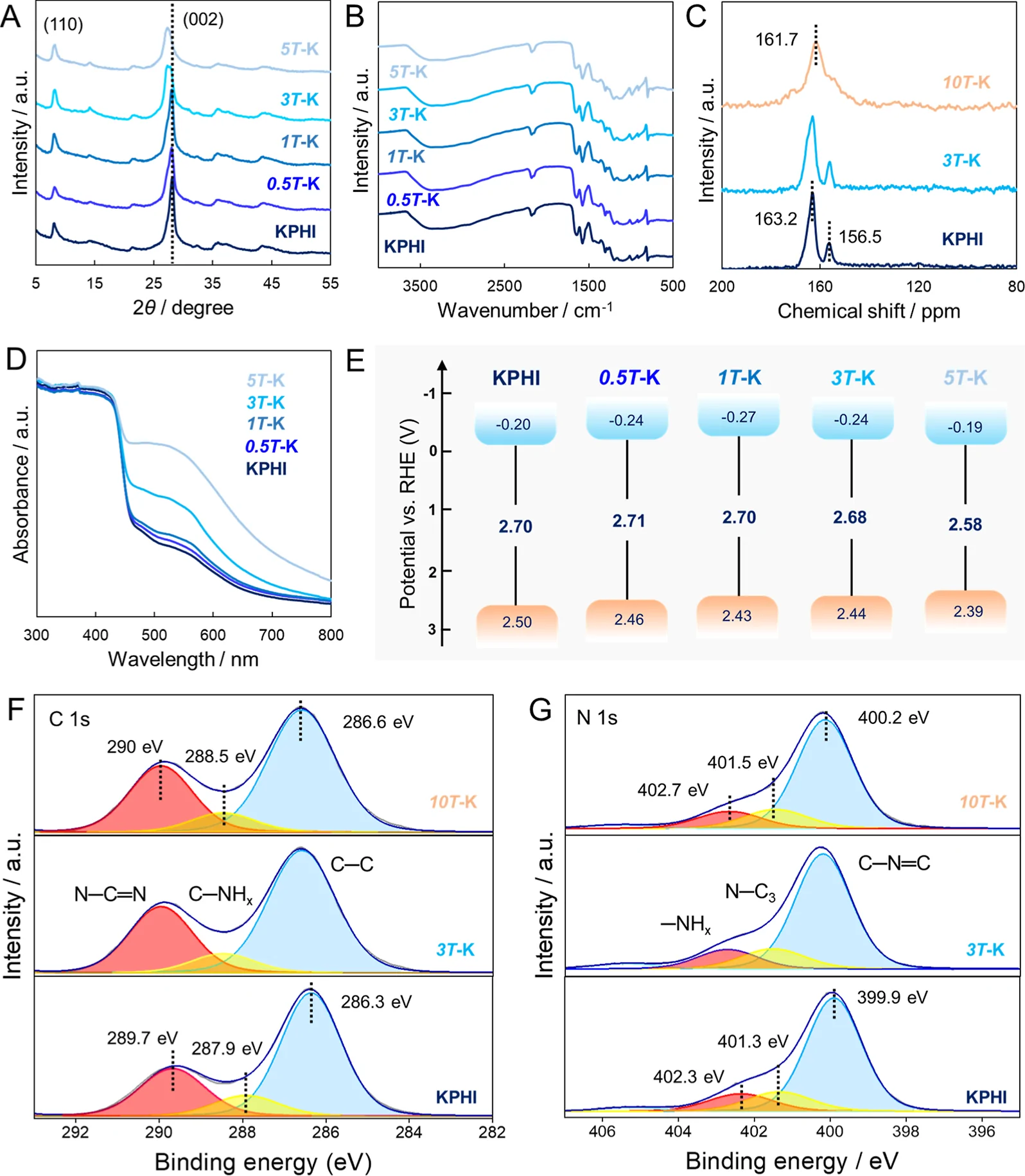
(A) XRD patterns, (B) FTIR spectra, and (C) Solid-state ^13^C CP-NMR of KPHI, 3T-K, and 10T-K. (D) UV–vis DRS spectra of KPHI and TAP-modified *K*PHI samples. (E) Band structures of all the catalysts. All potentials are reported versus the reversible hydrogen electrode (RHE) at pH 7. Conduction band positions were determined from Mott–Schottky measurements. Optical band gaps were estimated from UV–vis DRS using Tauc plot analysis, and valence band positions were calculated accordingly. High-resolution XPS spectra of (F) C 1s and (G) N 1s for KPHI, 3T-K, and 10T-K.

Elemental analyses revealed that with increasing TAP concentration, the C/N ratio shows a progressive increase from 0.59 for KPHI to 0.64 for 5T-K sample, signifying the successful carbon doping into the *K*PHI framework (Table S1). Simultaneously, a reduction in K content can be observed likely due to the interaction of TAP-derived decomposition byproducts with K species. These interactions may promote the volatilization of K-containing intermediates or interrupt their stable inclusion within the polymeric network, leading to an overall reduction in K retention within the final material. TAP-modified *K*PHI samples exhibit specific surface areas nearly identical to those of pristine KPHI (Figure S5 and Table S2). This finding suggests that surface area differences are unlikely to be a determining factor in their overall photocatalytic performance. Solid-state ^13^C cross-polarization nuclear magnetic resonance (CP-NMR) spectra reveal that both *K*PHI and 3T-K catalysts present two distinct peaks at 156.5 and 163.2 ppm. These peaks correspond to the characteristic carbon atoms in N=C–N_2_ (CN_3_) units of the heptazine ring and the sp^2^-hybridized N=C–N­(NH_*x*_), respectively (Figure 2c).^38^ However, higher concentration of TAP disrupted the polymeric framework, resulting in a shift of the CN_3_ peak to 161.7 ppm accompanied by a substantial attenuation and broadening of the CN_2_–(NH_*x*_) peak. UV–vis diffuse reflectance spectroscopy (DRS) was executed to assess whether TAP modulated the optical and electronic properties of the photocatalysts. As shown in Figure 2d, the TAP-modified *K*PHI samples exhibited a slight red-shift in the optical absorption edges, with the band gap (*E*_*g*_) gradually narrowed from 2.70 eV for KPHI to 2.58 eV for 5T-K. A closer inspection of the conduction band positions derived from Mott–Schottky measurements reveals a cathodic shift from −0.20 V vs. RHE for *K*PHI to −0.27 V for 1T-K (Figure S6). Similarly, the valence band potentials also shift cathodically, from 2.50 V for *K*PHI to 2.39 V for 5T-K, as shown in Figure 2e. These shifts suggest that the introduction of pyrimidine-like rings via carbon doping enables deliberate tuning of the PHI catalyst band structure, effectively extending its optical absorption range.

XPS analysis revealed notable changes upon TAP introduction (see Figure 2F,G). The high-resolution C 1s spectrum of pristine KPHI can be deconvoluted into three components at 286.3, 287.9, and 289.7 eV, corresponding to the adventitious carbon (C–C), edge-bound aminated carbons species (C–NH_*x*_) groups, and sp^2^ hybridized carbon (N–C=N) within the heptazine ring, respectively (Figure 2f). Similarly, the N 1s region shows three distinct peaks: bicoordinated N (C–N=C, N_2_C) at 399.9 eV, terminal amino groups (–NH_*x*_) at 401.3 eV, and tricoordinated N in the heptazine framework (N–C_3_, N_3_C) at 402.3 eV.^39,40^ Upon carbon doping both N 1s and C 1s core levels exhibit a consistent shift toward higher binding energies. This trend contrasts with most previous reports on covalent carbon nitrides, where carbon doping typically results in a shift to lower binding energies.^33–35^ While the incorporation of carbon forms pyrimidine-like rings, which are generally electron-rich, the observed shift indicates a reduction in local electron density around both N and C atoms. This contradiction can be associated with the substitution of N atoms with less electronegative carbon, altering the electronic structure of the heptazine units and overall electron redistribution. Additionally, the modified local bonding and possible interactions with K cations in the PHI framework further contribute to the electron deficiency, thereby increasing the binding energies of both N and C species. These findings suggest that while carbon doping enhances conjugation and optical properties, it also induces localized electronic perturbations that are reflected in the core-level XPS spectra. To further clarify the impact of pyrimidine modification, electron paramagnetic resonance (EPR) spectroscopy was carried out on KPHI and 0.5%T-KPHI samples (Figure S7). The spectra for both catalysts show a single Lorentzian signal, characteristic of delocalized unpaired π-electrons within the heptazine framework. 0.5%T-KPHI sample exhibited a stronger EPR signal relative to pure KPHI, suggesting an increased density of unpaired electrons.^22^ Taken together with complementary XRD, FTIR, elemental analysis, and solid-state NMR and EPR data, these findings support the conclusion that TAP serves as both a structural and electronic modifier.

### 3.2 Photocatalytic Performance

We explored the synthesis of 4-(pyrrolidin-1-yl)­benzonitrile through a photocatalytic strategy for C–N bond formation (Figure 3a), a transformation of high significance in pharmaceutical chemistry. Conventional protocols typically rely on palladium catalysts with sterically demanding ligands to drive reductive elimination. In contrast, Corcoran et al. introduced a paradigm shift by using Ni in conjunction with a photoactive cocatalyst capable of oxidizing the Ni center, thereby facilitating bond formation under photochemical conditions.^10^ Inspired by this advance, we designed a system employing TAP-modified KPHI as a heterogeneous, light-activated photocatalyst. The combination of KPHI, 4-bromobenzonitrile (1a), pyrrolidine (2a), DABCO, and NiBr_2_(dme) in DMA led to C–N coupling product 4-(pyrrolidin-1-yl)­benzonitrile (3a) in 73% yield under green (λ = 525 nm) LED irradiation (Figure 3b). Catalyst screening revealed that carbon doping markedly boosts the photocatalytic performance of KPHI, with the 0.5T-K variant achieving a 99% yield, representing nearly a 36% improvement over undoped KPHI. However, further increases in doping level (>0.5 wt %) led to reduced yields, likely due to increased defect density, disruption of the polymeric framework, and a decline in crystallinity, as supported by prior structural characterizations. These findings highlight that incorporating 0.5 wt % TAP strikes an optimal trade-off between preserving structural integrity and improving charge separation efficiency.

**Figure 3:**
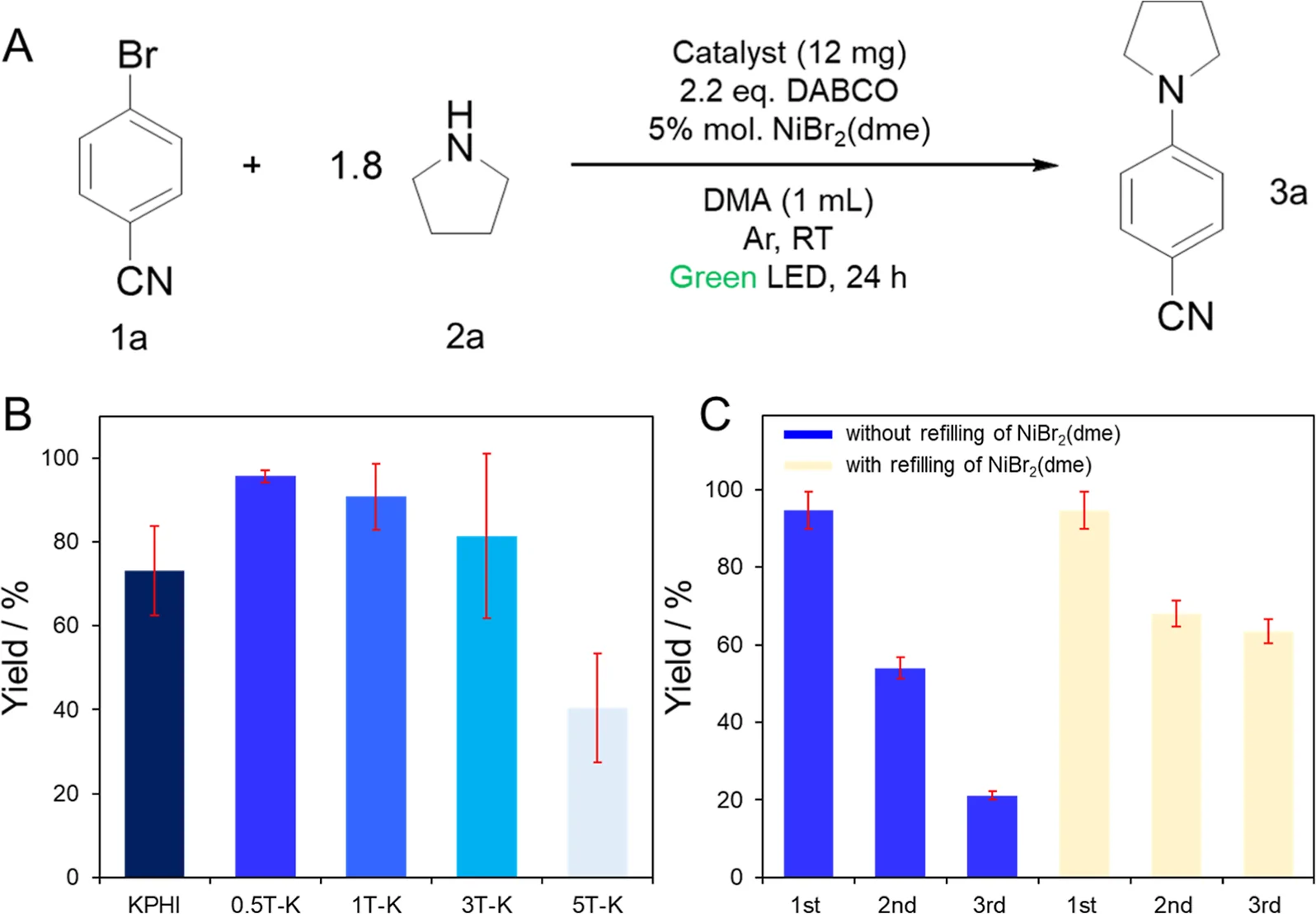
(A) Dual photoredox/nickel cross-coupling. (B) Product yield over various pristine KPHI and carbon-doped KPHI catalysts. (C) Recyclability tests with and without refilling of NiBr_2_(dme). Conditions: photocatalyst (12 mg), 1a (0.2 mmol), 2a (1.8 equiv), DABCO (2.2 equiv), NiBr_2_(dme) (5% mol.), DMA (1 mL), under Ar atmosphere with light irradiation for 24 h. Yields are calculated using GC-MS through calibration curve.

Control experiments further validated the photocatalytic nature of the reaction (Table S3). In the absence of light or the photocatalyst, no product formation was observed. Additionally, lack of NiBr_2_(dme) completely suppressed the reaction, emphasizing the essential roles of both the Ni cocatalyst and the photocatalyst in enabling the transformation. Notably, the use of DABCO proved critical as a proton shuttle, further contributing to the efficiency of the coupling reaction. The heterogeneous structure of 0.5T-K catalyst allows straightforward recovery and reuse. Following each photocatalytic run, the solid catalyst was collected, washed, and vacuum-dried for subsequent cycles. As illustrated in Figure 3c, the recycled catalyst without the addition of fresh NiBr_2_(dme) retained only 20% of its initial activity after the third cycle. The sharp decline in activity is understandable, as a portion of the Ni species likely leaches out during the washing of the spent catalyst, and the Ni catalyst is not directly anchored to the photocatalyst, further contributing to the loss. In comparison, supplementing with NiBr_2_(dme) helped to stabilize the performance, maintaining almost 70% of the original activity after three cycles. Previous studies suggest the catalyst deactivation in light-driven cross-coupling reactions may also result from the aggregation of Ni^0^ species or the formation of a Ni^I^–Ni^II^ dimer.^41,42^

With the optimized reaction conditions in hand, we first examined the scope of aryl halides in the C–N coupling reaction under visible-light irradiation (525 nm) at room temperature for 24 h (Table 1). Aryl bromides bearing electron-withdrawing substituents were efficiently converted to the desired amination products. Notably, 4-bromobenzonitrile delivered the corresponding product in excellent yield (1, 99%), demonstrating the high efficiency of the 0.5*T*-K/Ni catalytic system under mild conditions. Aryl bromides containing ester (2, 39%), methoxy (3, 47%), and acetyl groups (4, 31%) were also compatible, affording moderate yields. Electron-rich substrates were less reactive under the standard conditions. For example, methyl-substituted aryl bromide provided the product in lower yield (5, 8%), likely due to the slower oxidative addition of electron-rich aryl halides to the Ni^0^ species.

**Table 1:**
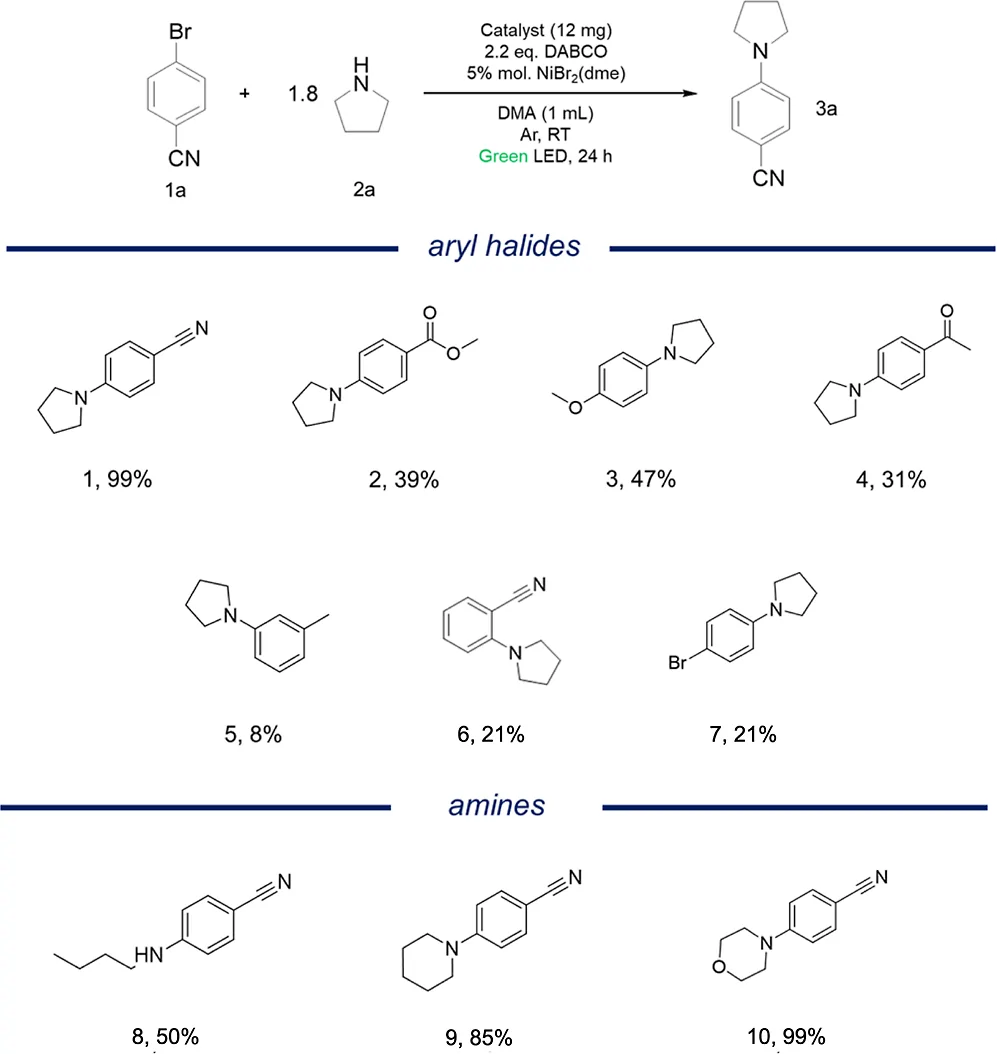
Scope of the Reaction Using 0.5KPHI as Photocatalyst

Standard conditions: photocatalyst (12 mg), 1a (0.2 mmol), 2a (1.8 equiv), DABCO (2.2 equiv), NiBr_2_(dme) (5% mol.), DMA (1 mL), under Ar atmosphere with light irradiation for 24 h.

Conversions and selectivity are calculated using GC-MS. NMR data for every product are included in the Supporting Information.

Substrates bearing sterically demanding or ortho-substituted groups were tolerated, although moderate yields were obtained (6 and 7, 21%), reflecting the increased steric hindrance during oxidative addition or reductive elimination steps. Next, we investigated the scope of amines using 4-bromobenzonitrile as the coupling partner. Secondary cyclic amines proved to be highly effective substrates. Piperidine and morpholine derivatives afforded the desired products in excellent yields (9, 85%; 10, 99%). An acyclic primary amine was also compatible, delivering the product in moderate yield (8, 50%). The successful coupling of primary amines is noteworthy, as such substrates are often challenging in Ni-catalyzed cross-coupling due to catalyst deactivation and competitive coordination. Overall, the substrate scope indicates that the 0.5T-K photocatalyst is particularly effective for electron-deficient aryl halides, while electron-rich substrates exhibit reduced reactivity, consistent with the known challenges in Ni-catalyzed oxidative addition. Although the scope remains moderate, these results demonstrate that electronically modulated PHI photocatalysts can promote C–N bond formation under mild green-light irradiation at ambient temperature. The ability to operate under low-energy visible light without external heating highlights the potential of this heterogeneous system as a sustainable alternative to conventional homogeneous photoredox catalysts, which typically require higher-energy light sources and elevated temperatures (Table S4).^43^

As it is well-known, scaling up photocatalytic reactions remains a major challenge due to issues such as poor light penetration, mass transfer limitations, and the need for uniform irradiation. These factors often hinder the translation of promising laboratory-scale protocols to more practical applications. To address this, we performed a scale-up of the optimized C–N coupling reaction by increasing the reaction volume 20-fold under otherwise identical conditions. Remarkably, the reaction proceeded with 44% yield after 24 h, demonstrating the robustness and scalability of our photocatalytic system based on 0.5T-K catalyst. After 96 h of reaction the final yield reached 58%, highlighting the potential of our heterogeneous photocatalyst for broader synthetic applications beyond small-scale batch processes. The XRD and FTIR analyses of the spent catalyst confirmed that the KPHI framework remained structurally intact postreaction, as no new peaks were observed (Figure S8). Nonetheless, a reduction in the intensity of the NH_*x*_ and cyano group signals was detected possibly due to their involvement in the reaction. High-angle annular dark-field scanning TEM imaging revealed that the rod-like morphology of KPHI was preserved, although nanoparticles were observed surrounding the crystals (Figure S9). Elemental mapping indicated that these nanoparticles are composed of Ni species, which appear to be partially oxidized given the overlapping O and Ni signals. The aggregation and oxidation of these Ni species likely contributes to the decline in catalytic activity over successive cycles in small-scale batch experiments, as shown in Figure 3C.

To further clarify the enhanced performance occurring over TAP-modified PHI catalysts, a series of photo­(electro)­chemical measurements were carried out. As previously discussed, the incorporation of TAP during thermal polymerization leads to extended optical absorption and slightly narrowed band gaps, indicating that 0.5T-K possesses superior light absorption capacity under our reaction conditions. Upon excitation at 365 nm, pure KPHI exhibits a major photoluminescence (PL) emission at 500 nm, which is associated with the radiative recombination of photogenerated charge carriers (Figure 4a). This emission is gradually quenched with increasing TAP content, indicating that the introduction of pyrimidine-like moieties promotes more effective charge separation in 0.5T-K. Time-resolved PL decay analysis supports this finding, where 0.5T-K catalyst shows a shorter average lifetime (1.02 ns) relative to pristine KPHI (1.5 ns) (see inset of Figure 4a), demonstrating a suppression of radiative recombination due to electronic structure modulation induced by carbon doping. Electrochemical impedance spectroscopy (EIS) shows reduced charge transfer resistance for 0.5T-K, as evidenced by its smaller Nyquist semicircle, signifying improved interfacial charge transfer (Figure 4c). Complementary transient photocurrent measurements reveal a greater density of photogenerated carriers and enhanced current response for 0.5T-K compared to pure KPHI (Figure 4b). Altogether, these favorable electronic and charge dynamics correlate well with the enhanced photocatalytic activity of 0.5T-K catalyst in C–N cross-coupling reactions.

**Figure 4:**
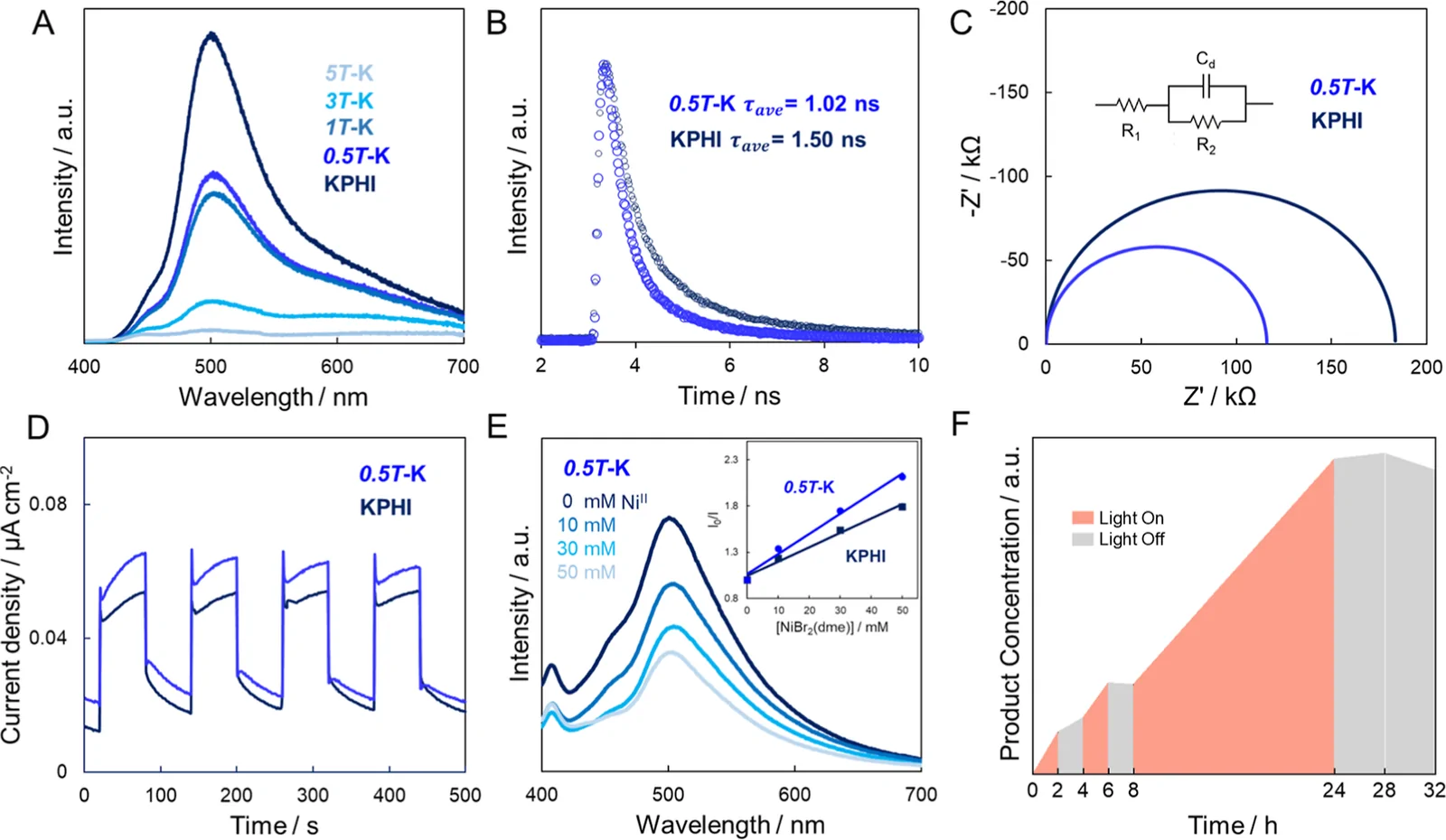
(A) Room temperature steady-state PL emission spectra of KPHI and *TAP*-modified KPHI catalysts with an excitation wavelength of λ = 380 nm. (B) Solid-state time-resolved PL decay of KPHI and 0.5T-K. (C) EIS Nyquist plots. (D) Transient photocurrent (λ = 525 nm) for KPHI and 0.5T-K in 0.2 m Na_2_SO_4_ aqueous solution. (E) Steady-state emission quenching of 0.5T-K with varying NiBr_2_(dme) concentrations. (F) Time profile of product concentration (3a) with and without light irradiation.

To get mechanism insight, a series of control experiments were conducted (Table S3). The complete suppression of product formation upon addition of AgNO_3_ confirms the involvement of electrons in the catalytic cycle. Similarly, the absence of conversion in the presence of triethanolamine highlights the essential role of photogenerated holes. The reaction was entirely inhibited by 2,2,6,6-tetramethylpiperidine 1-oxyl, indicating the participation of radical intermediates. To probe the interaction between the photocatalysts and Ni species, the steady-state emission quenching experiments were performed. Progressive addition of NiBr_2_(dme) led to efficient quenching of the excited state of 0.5*T*-K, affording a linear Stern–Volmer relationship (Figures 4e, and S10). The quenching constant of 0.5*T*-K toward NiBr_2_(dme) is greater than that of KPHI, suggesting more efficient charge transfer. This enhanced quenching behavior is consistent with stronger electrostatic interactions between Ni^II^ and 0.5*T*-K, facilitating photoreduction of the Ni species. The light dependence of the transformation and exclusion of a radical chain pathway were verified by light on/off experiments, in which no product formation was observed in the dark (Figure 4f).

Collectively, these findings, together with literature precedents, support a dual photoredox/Ni catalytic mechanism for the C–N bond formation,^44,45^ as illustrated in Figure 5. Upon visible-light irradiation, excitation of 0.5T-K occurs concurrently with the reduction of Ni^II^ to active Ni species (Ni^I^/Ni^0^). This is followed by oxidative addition of the aryl halide to the Ni^0^ center, generating a Ni^II^ intermediate. Simultaneously, photogenerated holes oxidize pyrrolidine to produce a pyrrolidine radical. This radical intermediate can undergo deprotonation in the presence of DABCO, thereby facilitating further formation of pyrrolidine radicals. The Ni^II^ intermediate then coordinates with the pyrrolidine radical, yielding a Ni^III^ complex. The desired coupling product is obtained by reductive elimination from this Ni^III^ species. Finally, electron transfer from photoexcited 0.5T-K to the resulting Ni^I^ species regenerates both the active Ni^0^ catalyst and DABCO, completing the dual catalytic cycle. In this mechanism, DABCO plays a key role in promoting pyrrolidine radical generation and sustaining the overall catalytic process.

**Figure 5:**
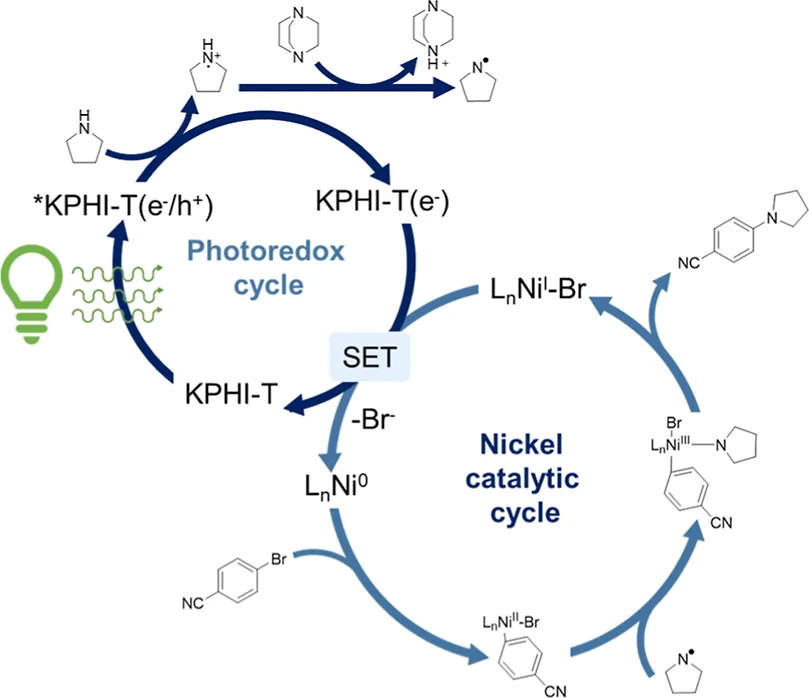
Proposed mechanism of photoredox C–N coupling on KPHI catalysts.

## 4 Conclusion

In summary, we developed a strategic approach to boost the green light-driven C–N coupling reaction performance of ionic carbon nitrides. Incorporating TAP during polymerization induced carbon doping of the PHI framework, enabling precise band structure modulation. The optimal catalyst (0.5T-K) reached up to 99% conversion of 4-bromobenzonitrile to 4-(pyrrolidin-1-yl)­benzonitrile, with a 58% yield obtained in prolonged 96 h scale-up reaction. This approach demonstrates compatibility with a range of aryl halides and amines, with yields depending on substrate electronic properties. Comprehensive characterizations indicated that carbon doping promotes C–N cross-coupling reaction by extending the solar coverage, tuning the electronic structure, and facilitating charge transport. Overall, this work highlights the potential of ionic carbon nitrides as efficient, low-cost, and sustainable platforms for photocatalytic C–N formation in organic synthesis.

## Supplementary Material



## Notes

Detailed data of the materials characterizations, SEM and TEM images, elemental analysis, thermogravimetric analysis, BET measurements, Mott–Schottky plots, EPR, and NMR spectra of the products (PDF)

## #

Institute of Chemistry, University of Potsdam, Potsdam 14476, Germany
