# Supplementary material for: Pyrimidine-Mediated Electronic Modulation of Ionic Carbon Nitrides for Green Light-Driven Photocatalysis
Source: ACS Mater Au. 2026 Jul 3;6(5):1131–40. doi: 10.1021/acsmaterialsau.6c00080 (PMC13564264; doi:10.1021/acsmaterialsau.6c00080)

Supporting Information

Pyrimidine-Mediated Electronic Modulation of Ionic Carbon Nitrides for Green Light-Driven Photocatalysis

Anna Lo Presti,[1] Serge Arnold Benedoue,[2] Sonia Zoltowska,[1] Sue-Faye Ng,[1] Sergio Navalón,[3] and Christian Mark Pelicano[*, 1]*

[1] Department of Colloid Chemistry, Max Planck Institute of Colloids and Interfaces,Potsdam 14476, Germany

[2] Department of Inorganic Chemistry, Faculty of Science, University of Yaoundé 1, P.O. Box 812, Yaoundé 812, Cameroon

[3]Department of Chemistry, Universitat Politècnica de València, C/Camino de Vera, s/n, 46022,Valencia, Spain

†A. Lo Presti and S. A. Benedoue contributed equally to this work.

Corresponding author: christianmark.pelicano@mpikg.mpg.de

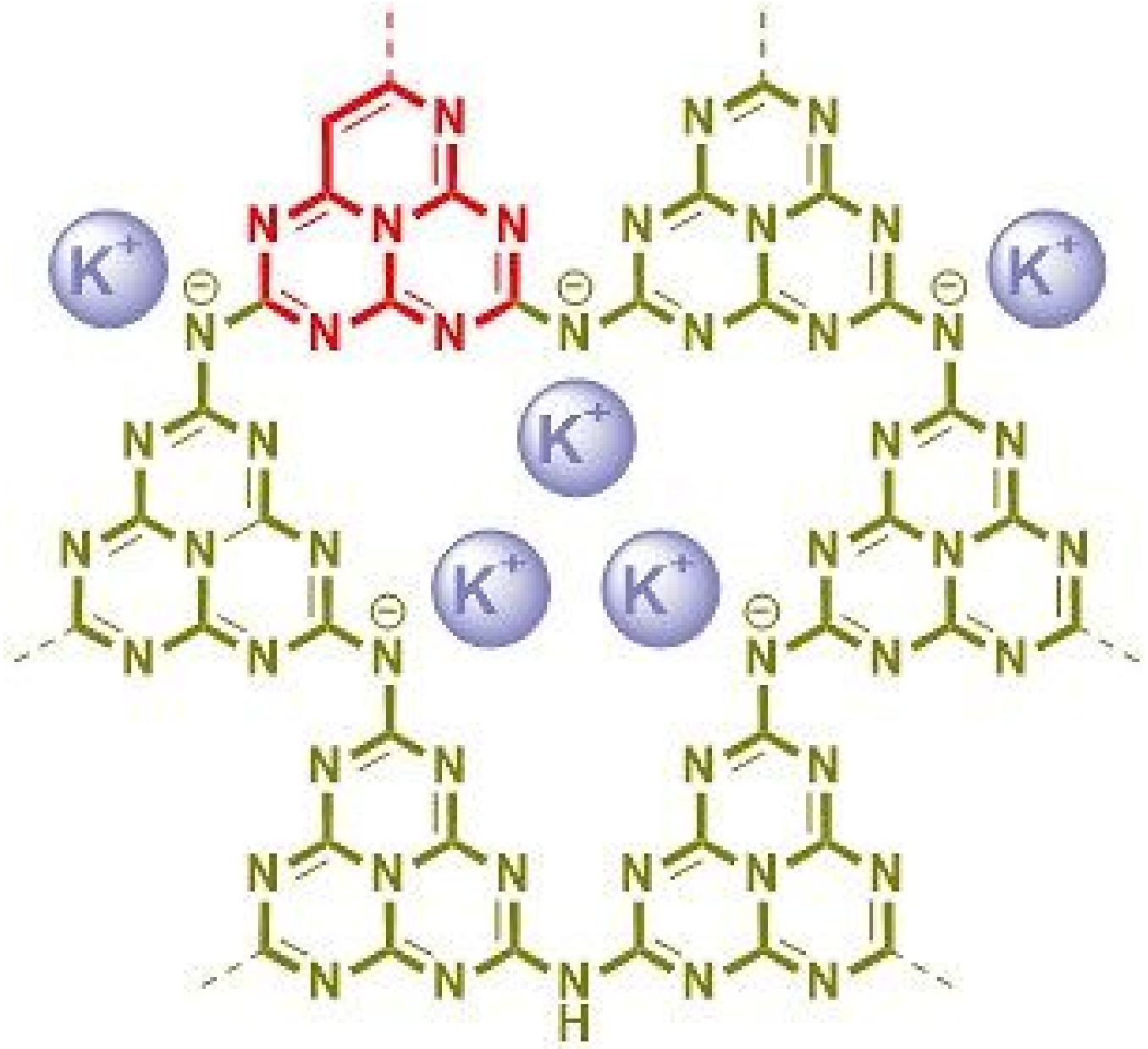

Figure S1. Structure of pyrimidine-doped poly(heptazine imide) catalyst.

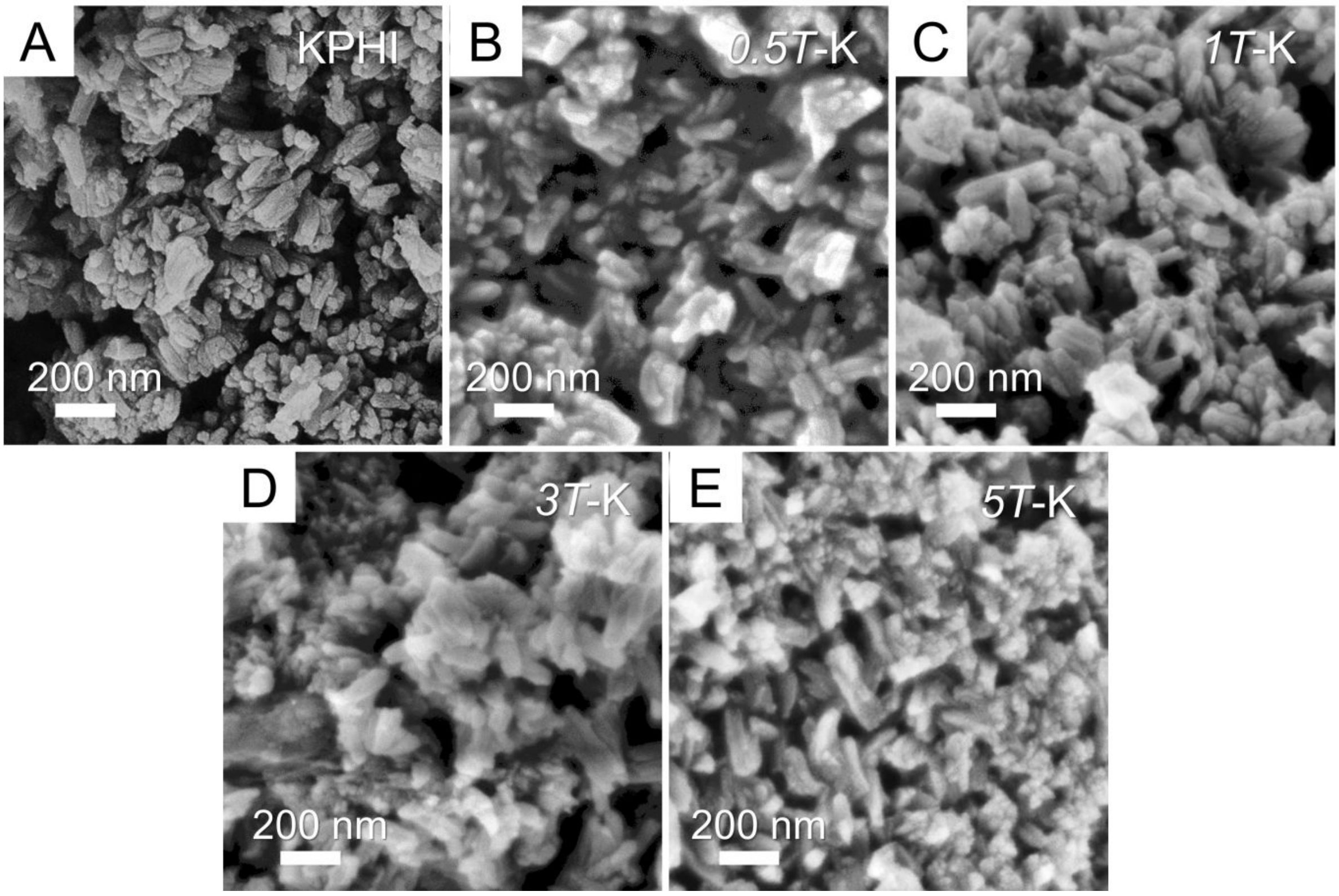

Figure S2. SEM images of A) KPHI, B) *0.5T*-K, C) *1T*-K, D) *3T*-K, and E) *5T*-K catalysts.

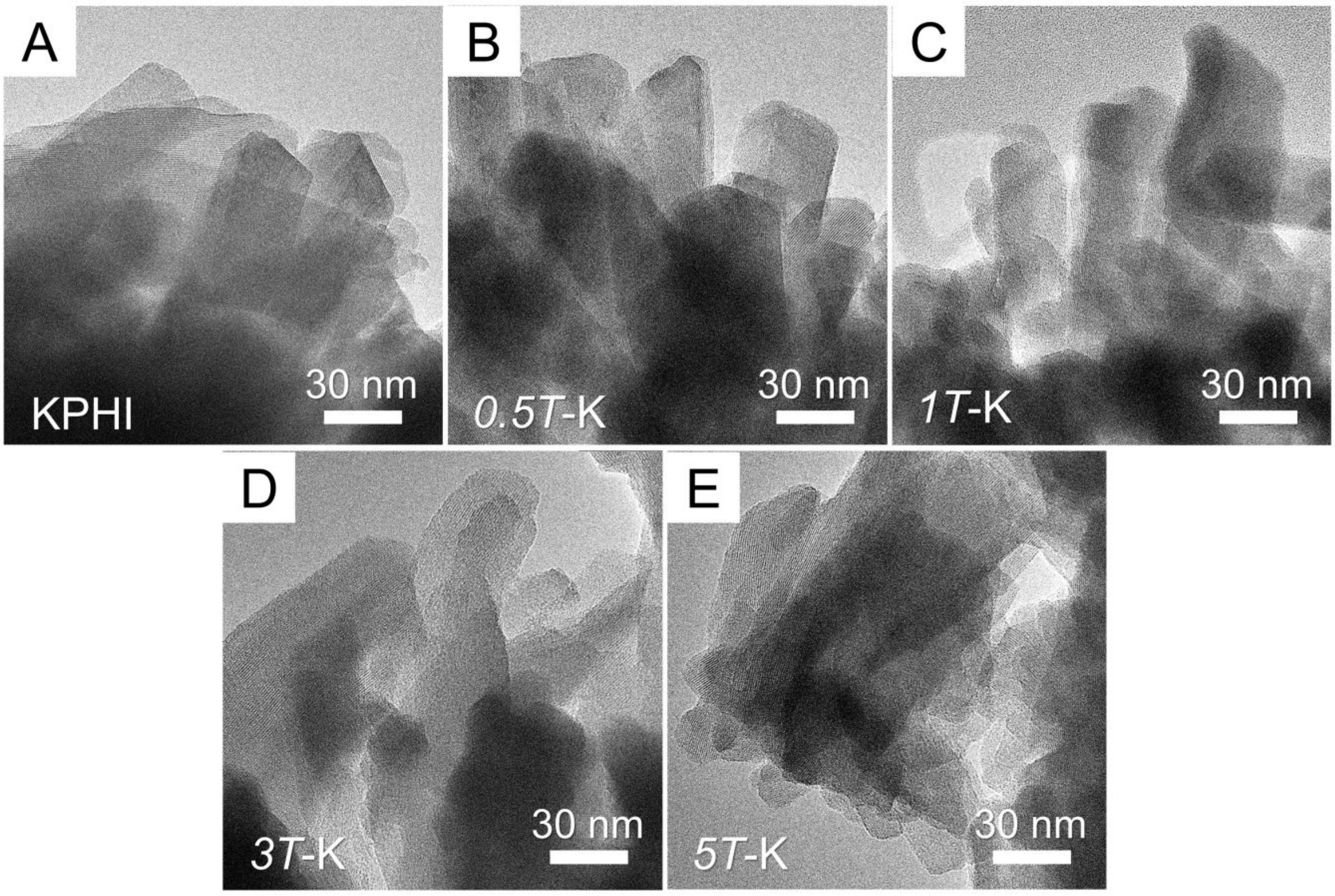

Figure S3. High-resolution TEM images of A) KPHI, B) *0.5T*-K, C) *1T*-K, D) *3T*-K, and E) *5T*-K catalysts.

Table S1. The relative ratios of C, N, H and O elementals in all samples determined by elemental analysis and the total amount of alkali metals in all catalysts via ICP.

**Samples**	**C (%)**	**N (%)**	**C/N**	**H (%)**	**O (%)**	**K (%)**	**Li (%)**
KPHI	27.3	45.9	0.59	2.3	14.5	12.4	0.2
0.5T-K	28.3	46.5	0.61	2.2	13.8	10.6	0.2
1T-K	28.7	46.1	0.62	2.2	13.2	9.1	0.2
3T-K	28.0	45.8	0.61	2.2	13.1	7.8	0.2
5T-K	28.6	44.9	0.64	2.3	13.2	8.4	0.2

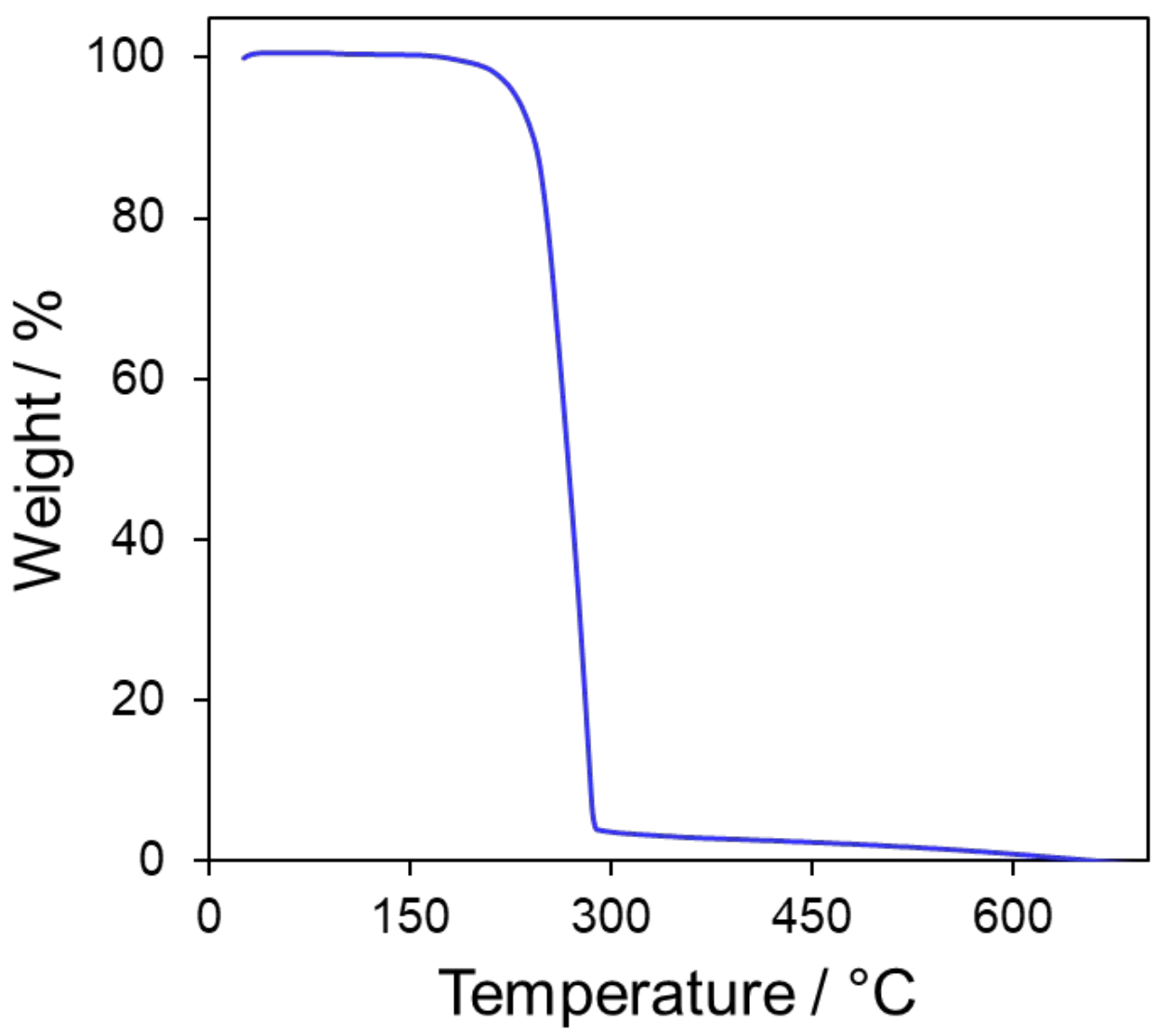

Figure S4. TG curve of TAP recorded under N_2 atmosphere (10 K min^{-1}).

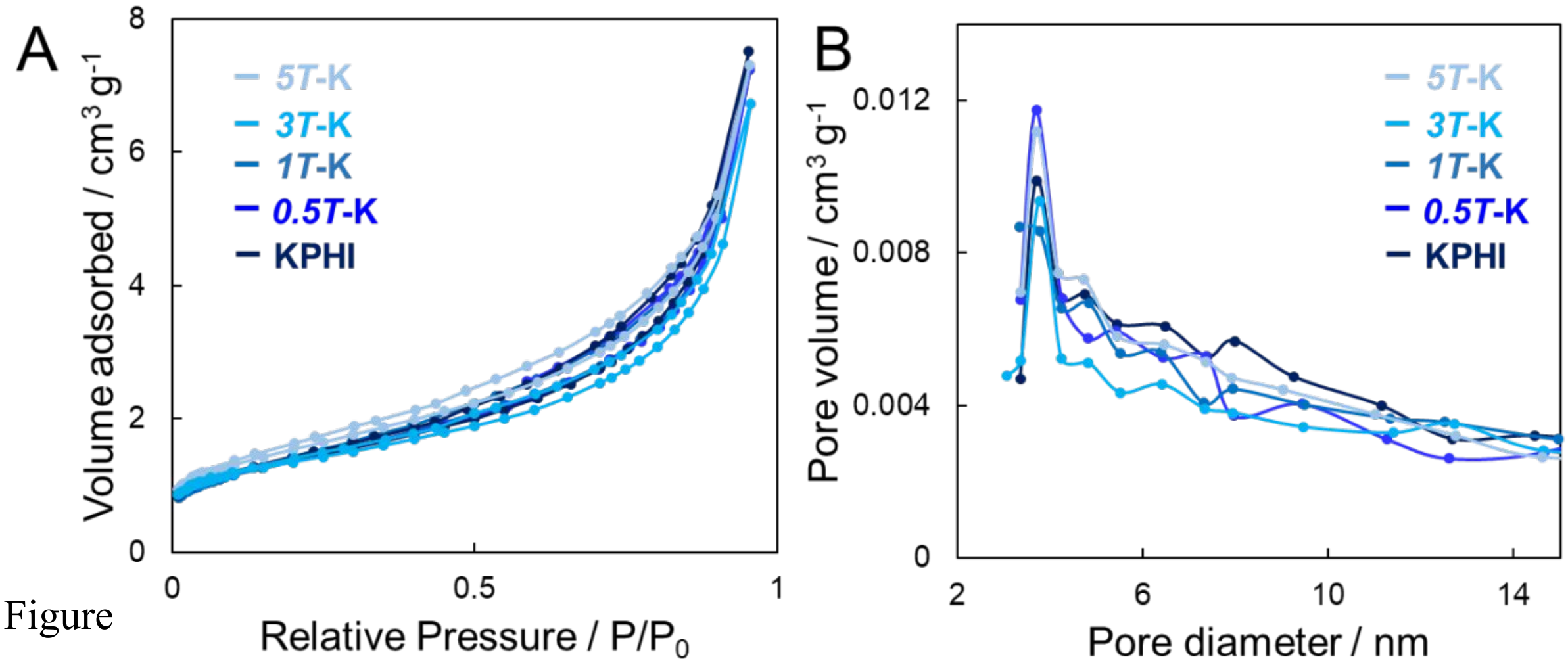

Figure S5. TG curve of TAP recorded under N_2 atmosphere (10 K min^{-1}).

Table S2. BET specific area pore volume and diameter size of all prepared samples.

Sample	S_{BET} $m^2\ g^{-1}$	V_p $cm^3\ g^{-1}$	D_p nm
KPHI	44.6	0.07	3.7
0.5T-K	43.7	0.06	3.7
1T-K	43.8	0.06	3.3
3T-K	45.7	0.06	3.8
5T-K	51.8	0.07	3.7

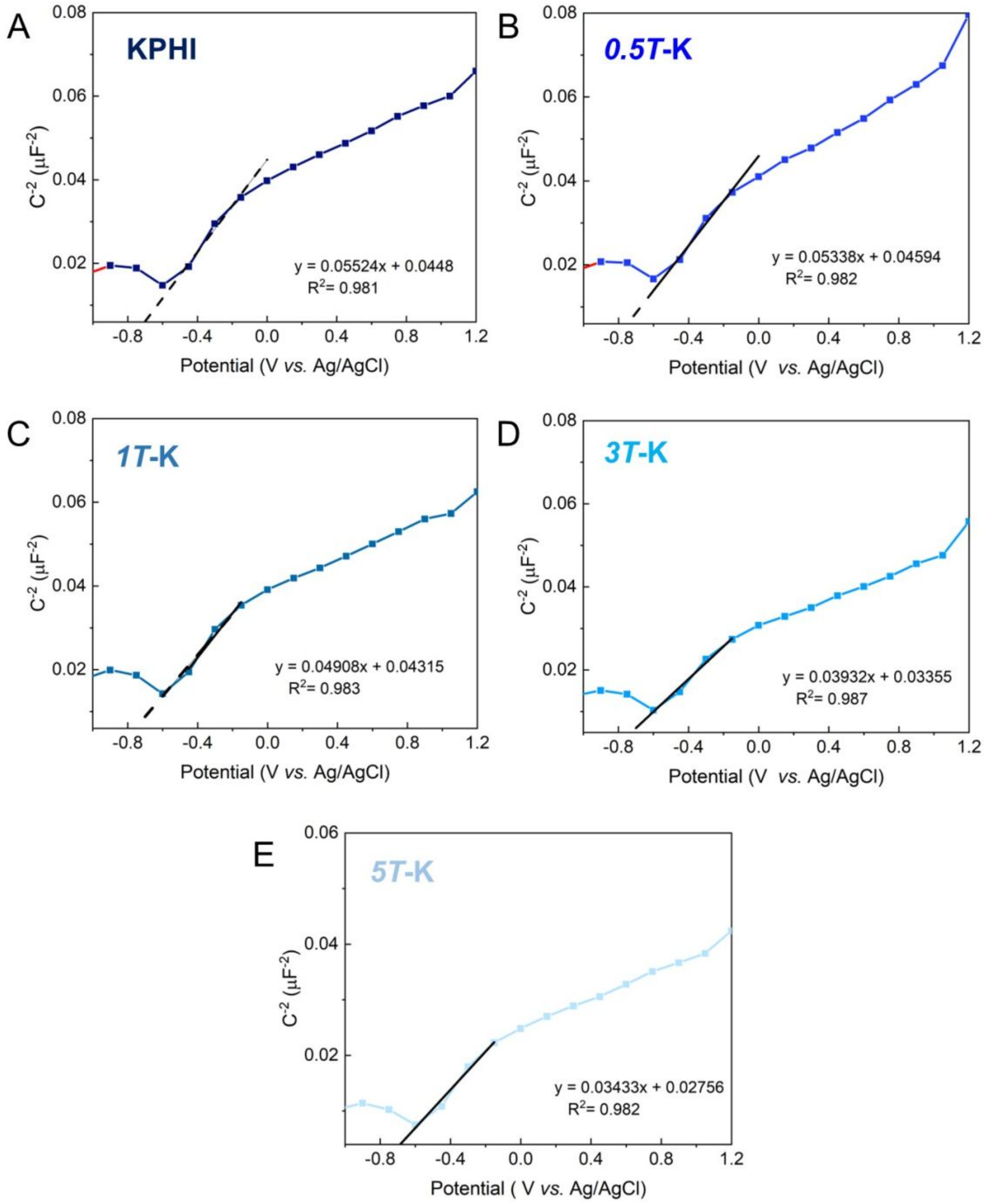

Figure S6. Mott-Schottky plots of all the catalysts.

Table S3. Control experiments on the model reaction[a]

Deviation	Yield of **3a**[b] / %
None	96
No LED light	n.d.[c]
No photocatalyst	n.d.
No DABCO	n.d.
No NiBr2(dme)	n.d.
+$AgNO_3$	n.d.
+TEOA	n.d.
+TEMPO	n.d.

[a]Conditions: photocatalyst (12 mg), 1a (0.2 mmol), 2a (1.8 eq), DABCO (2.2 eq), $NiBr_2$(dme) (5% mol.), DMA (1 mL), $AgNO_3$ (0.2 mmol), TEOA (0.2 mmol), TEMPO (0.2 mmol) under Ar atmosphere with light irradiation for 24 h.

[b]Yields are calculated using GC-MS through calibration curve.

[c]Not detected

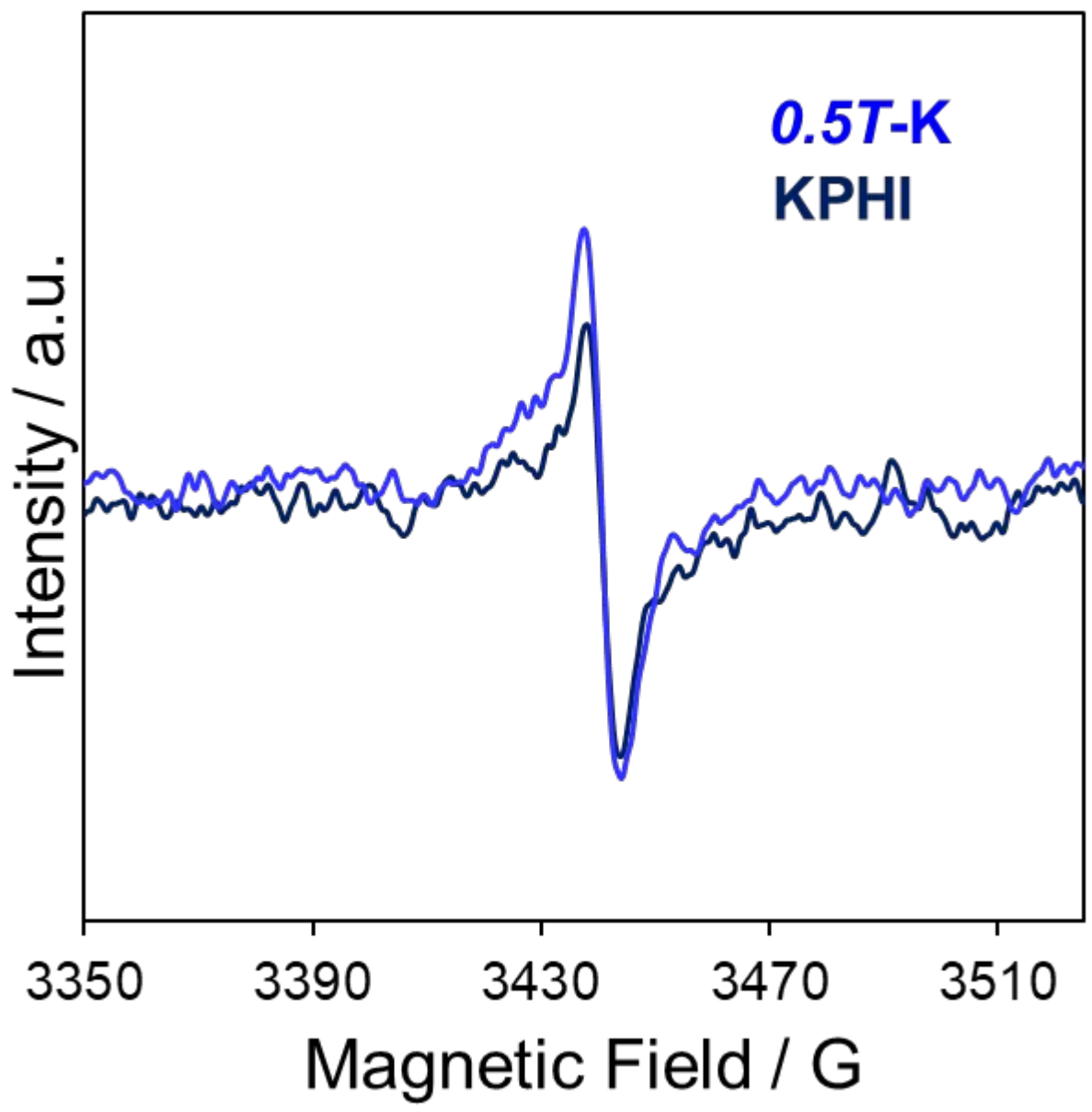

Figure S7. Solid-state EPR spectrum of KPHI and *0.5T*-K.

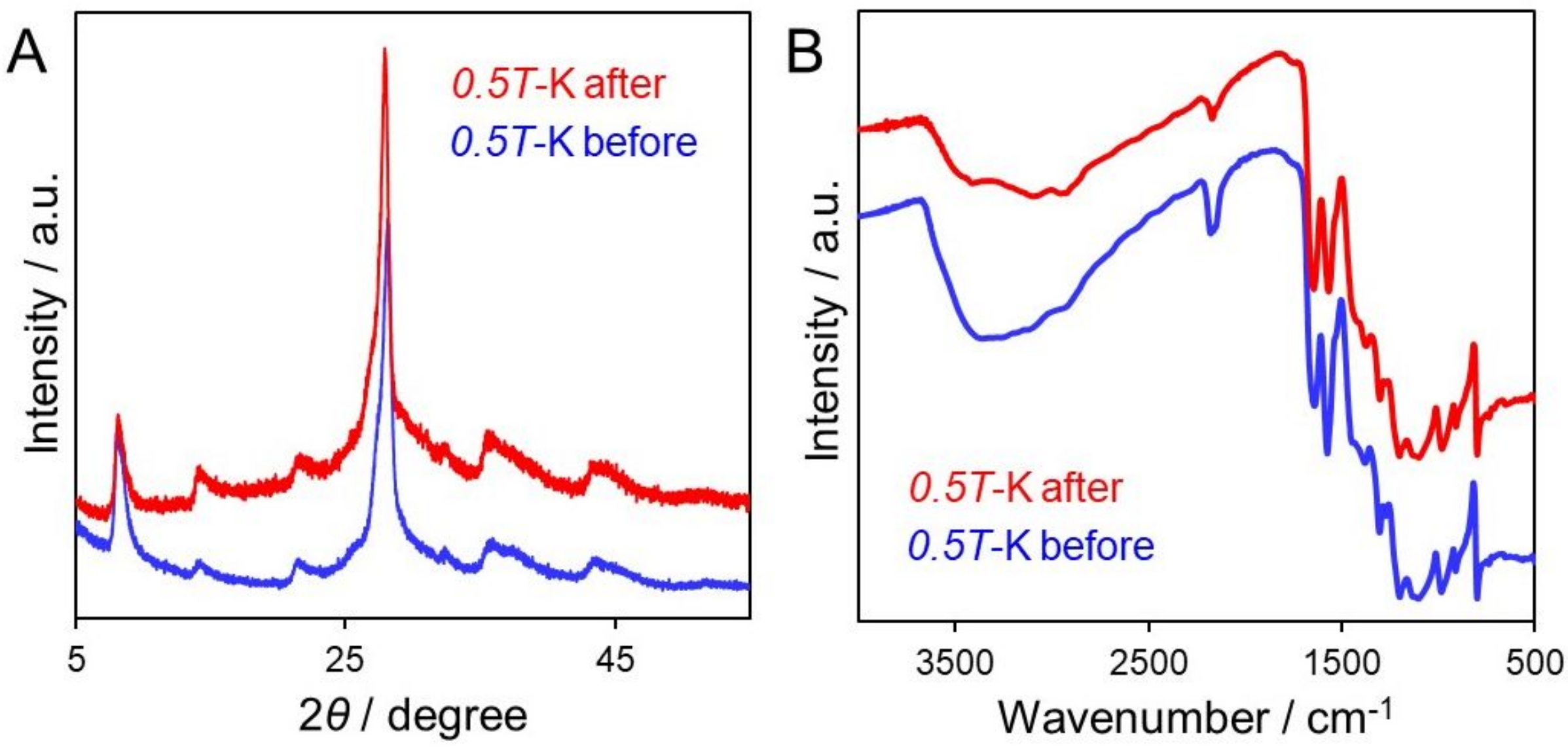

Figure S8. A) XRD patterns and B) FTIR spectra of 0.5T-K catalyst before and after large-scale reaction.

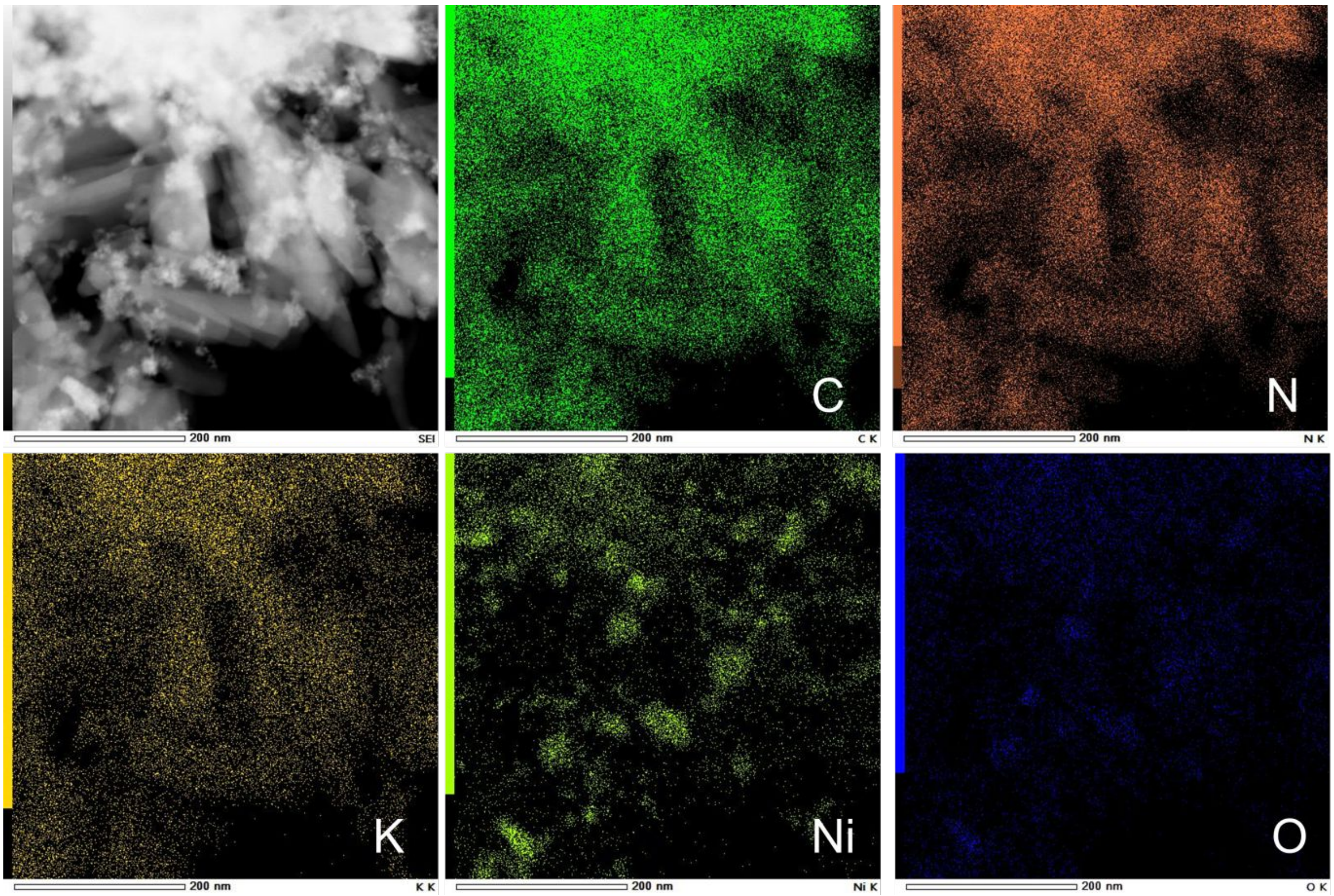

Figure S9. HAADF–STEM image of 0.5T-K catalyst after large-scale reaction and its correponding elemental mapping images.

Table S4. Comparison of reported photocatalytic C–N cross-coupling catalysts and reaction conditions.

Catalyst	Type	Light Source (nm)	T (°C)	Time (h)	Notes	Reference
Ir[dF(CF_3)ppy]$_2$(dtbbpy)PF_6	Homo	34 W Blue-LED	RT	24	Dual Ir/Ni, tolerant, O_2 compatible	*Angew. Chem. Int. Ed.* 2016, 55, 13219–13223.
Ru(bpy)$_3$(PF_6)$_2$	Homo	3 W 400 nm-LED (x2)	RT	24	Radical–radical C(sp^3)–N coupling; inert atmosphere	*ACS Catal.* 2021, 11, 9, 5026–5034.
Benzophenone derivative (PC1) / Thioxanthone (TXO)	Homo	395 nm LED	30 (50 °C for Ar–Cl)	12	Divergent C–C / C–N coupling; 10–20 mol% PC	*Org. Lett.* 2025, 27, 32, 9083–9089.
Ir polypyridyl complex	Homo	450 nm - LED	RT	36	Ni/Ir dual photoredox C–N coupling	*J. Am. Chem. Soc.* 2020, 142, 37, 15830–15841.
4CzIPN	Homo	30 W 450 nm-LED	18–25	12	Requires $NiBr_2$(dme), dtbpy ligand	*Org. Chem. Front.* 2021, 8, 4118-4123
Ni/MCN-B	Hetero	50 W 420 nm-LED	RT	12	Integrated Ni (5-7 wt%)–carbon nitride catalyst for C–N coupling	*J. Catal.* 2024, 433, 115461.
CuI/PCN	Hetero	20 W 420 nm-LED	RT	10–24	Good–excellent yields and recyclable photocatalyst	*Sci. Bull.* 2022, 67, 71.
NV-P-C_3N_4 + Ni salt	Hetero	450 nm-LED	RT	48	High yields (75–97%) but requires long irradiation time	*Chem. Catal.* 2022, 2, 3546.
Ni–Ir@TpBpy COF	Hetero	427 nm-LED	40	24	Yields up to 94%; with Ir and elevated temperature	*J. Am. Chem. Soc.* 2022, 144, 17, 7822–7833.
Ni@PQCOF	Hetero	456 nm-LED	RT	24	Multiple C(sp^2)–X couplings (C–N, C–S, C–O, C–C, etc.); requires blue light	*Nat. Commun.* 2025, 16, 4716.
Ni-mpg-CNx	Hetero	447 nm-LED	60	24	Broad functional group tolerance	*Angew. Chem. Int. Ed.* 2022, *61*, e202203176
Ni/Carbon nitride	Hetero	450 nm-LED	55 °	20	Single atom Ni on CN	*Chem. Sci.*, 2022,13, 8536-8542
CN-OA-m	Hetero	525 nm	RT	24	Same conditions Yield: 87%	*Solar RRL,* 2023, 7 2300077
bulk-CN	Hetero	525 nm	RT	24	Control experiment Yield: 30%	***This work***
0.5T-K	Hetero	525 nm	RT	24	Compatibility with electronically diverse aryl halides and amines under mild green-light irradiation Yield: 96%	***This work***

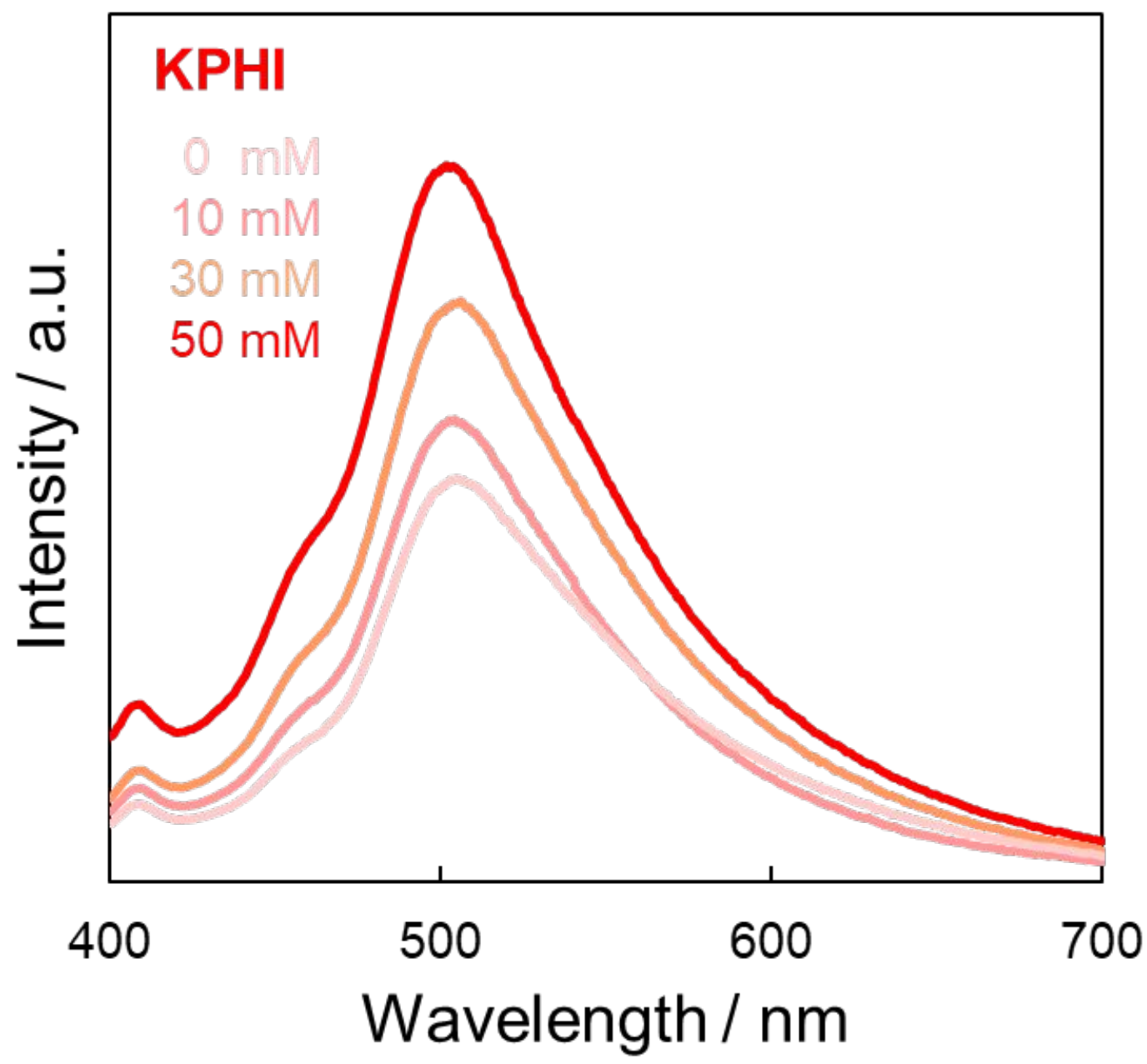

Figure S10. Steady-state emission quenching of KPHI with varying $NiBr_2$(dme) concentrations.

Characterization for C-N coupling

1 {4-(pyrrolidin-1-yl)benzonitrile}

^{1}H NMR (400 MHz, CDCl3) δ: 7.43 (2H, J = 8.8 Hz, d), 6.49 (2H, J = 8.8 Hz, d), 3.33-3.30 (4H, m), 2.05-2.01 (4H, m)

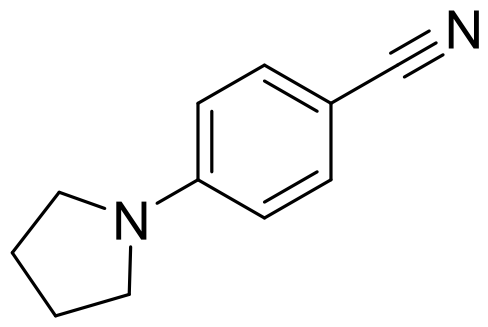

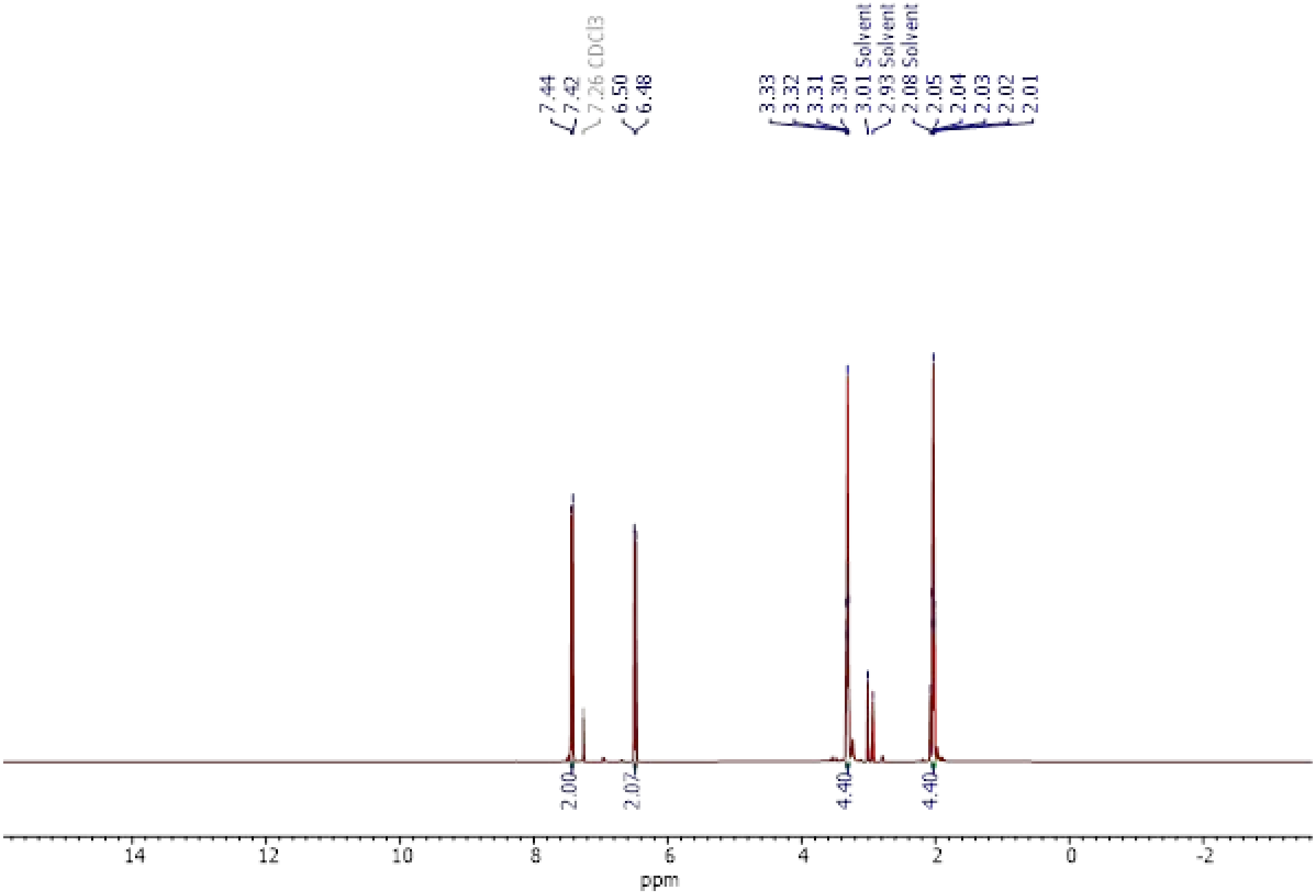

2 { methyl 4-(pyrrolidin-1-yl)benzoate}

^{1}H NMR (400 MHz, CDCl3) δ: 7.81 (2H, J = 8.9 Hz, d), 6.42 (2H, J = 8.9 Hz, d), 3.77 (3H, s), 3.28-3.25 (4H, m), 1.97-1.90 (4H, m)

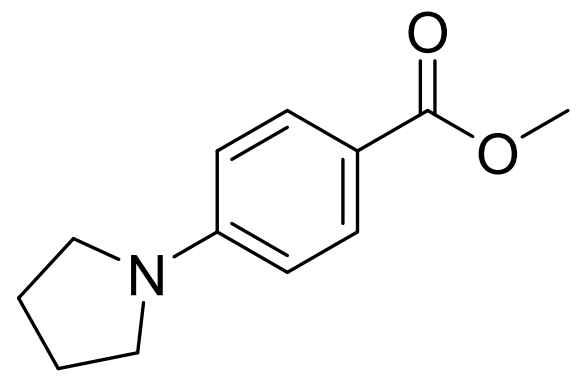

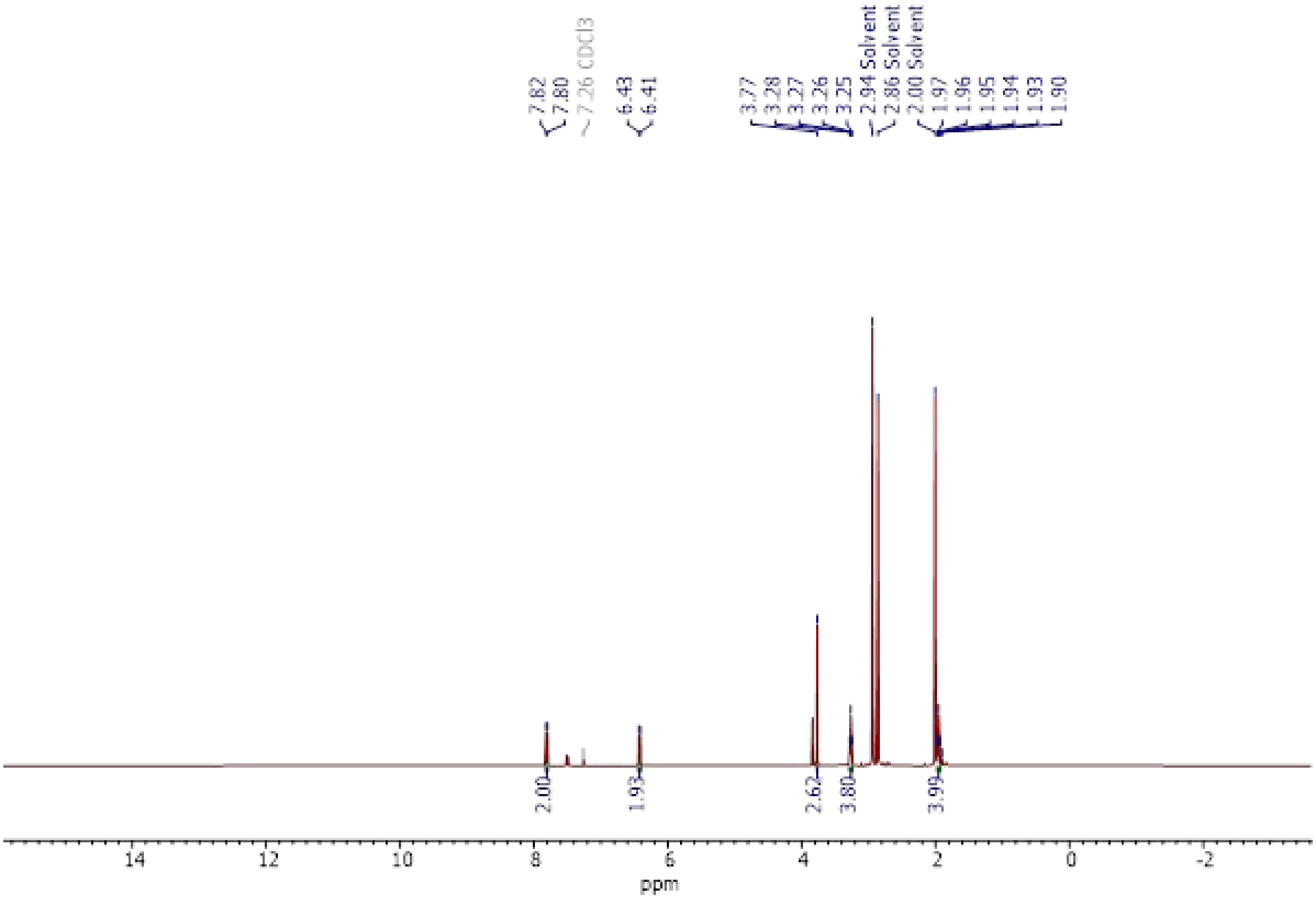

3 { 1-(4-methoxyphenyl)pyrrolidine}

^{1}H NMR (400 MHz, $CDCl_3$) δ:6.85 (2H, J = 8 Hz, d), 6.68 (2H, J = 8 Hz, d), 3.77 (3H, s), 3.28-3.25 (4H, m), 2.03-1.99 (4H, m)

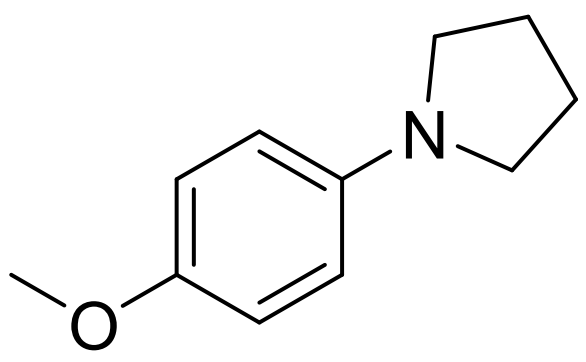

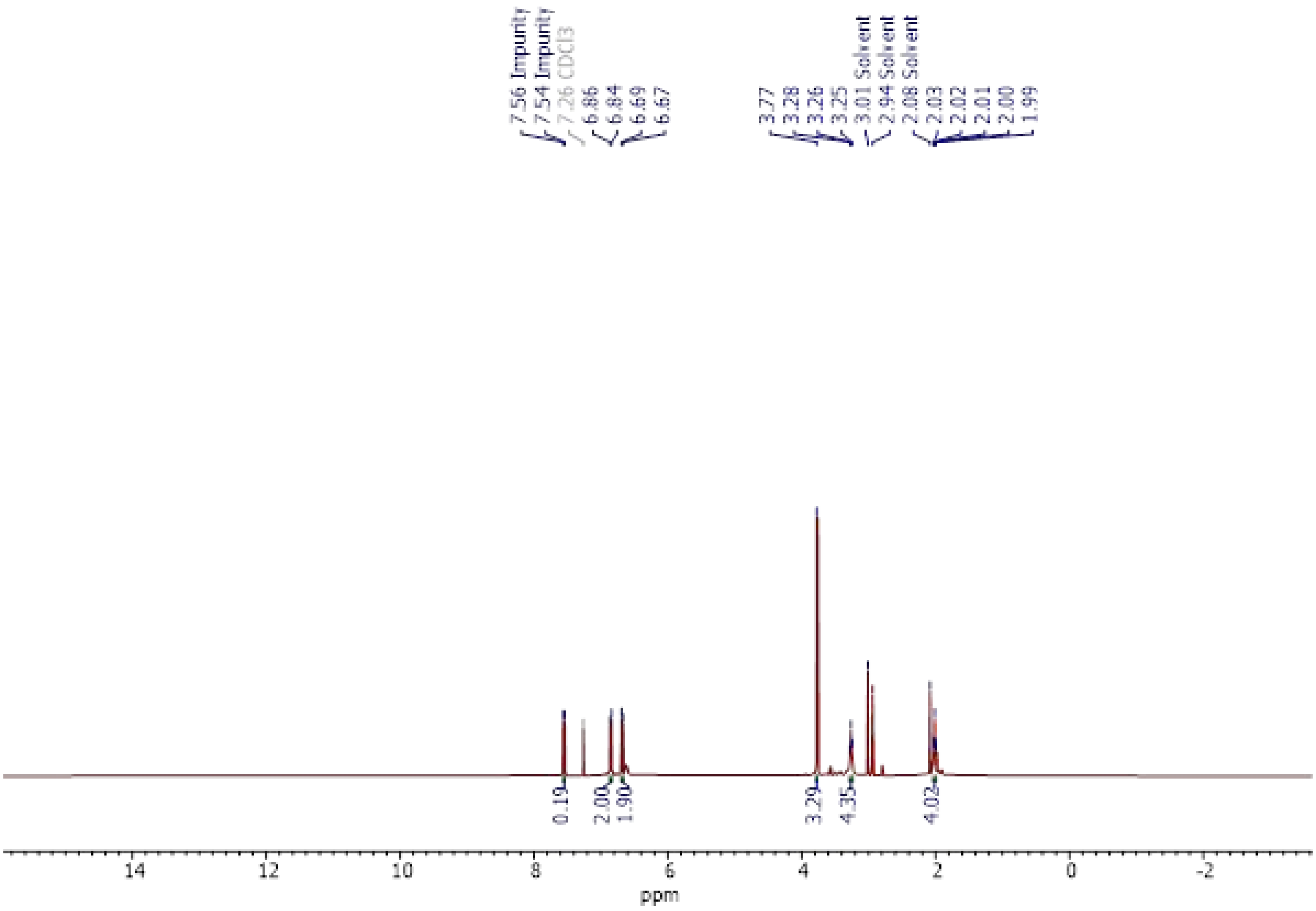

4 [1-(4-(pyrrolidin-1-yl)phenyl)ethan-1-one]

^1H NMR (400 MHz, $CDCl_3$) δ: 7.85 (2H, J = 8 Hz, d), 6.50 (2H, J = 8 Hz, d), 3.37-3.33 (4H, m), 2.49 (3H, s), 2.04-2.01 (4H, m)

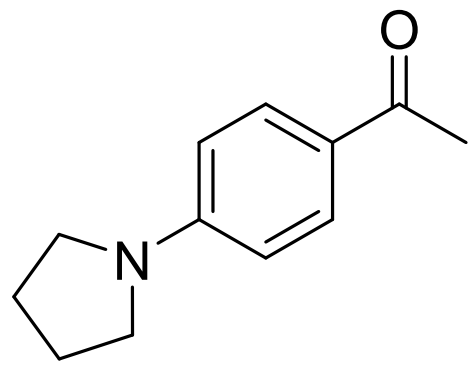

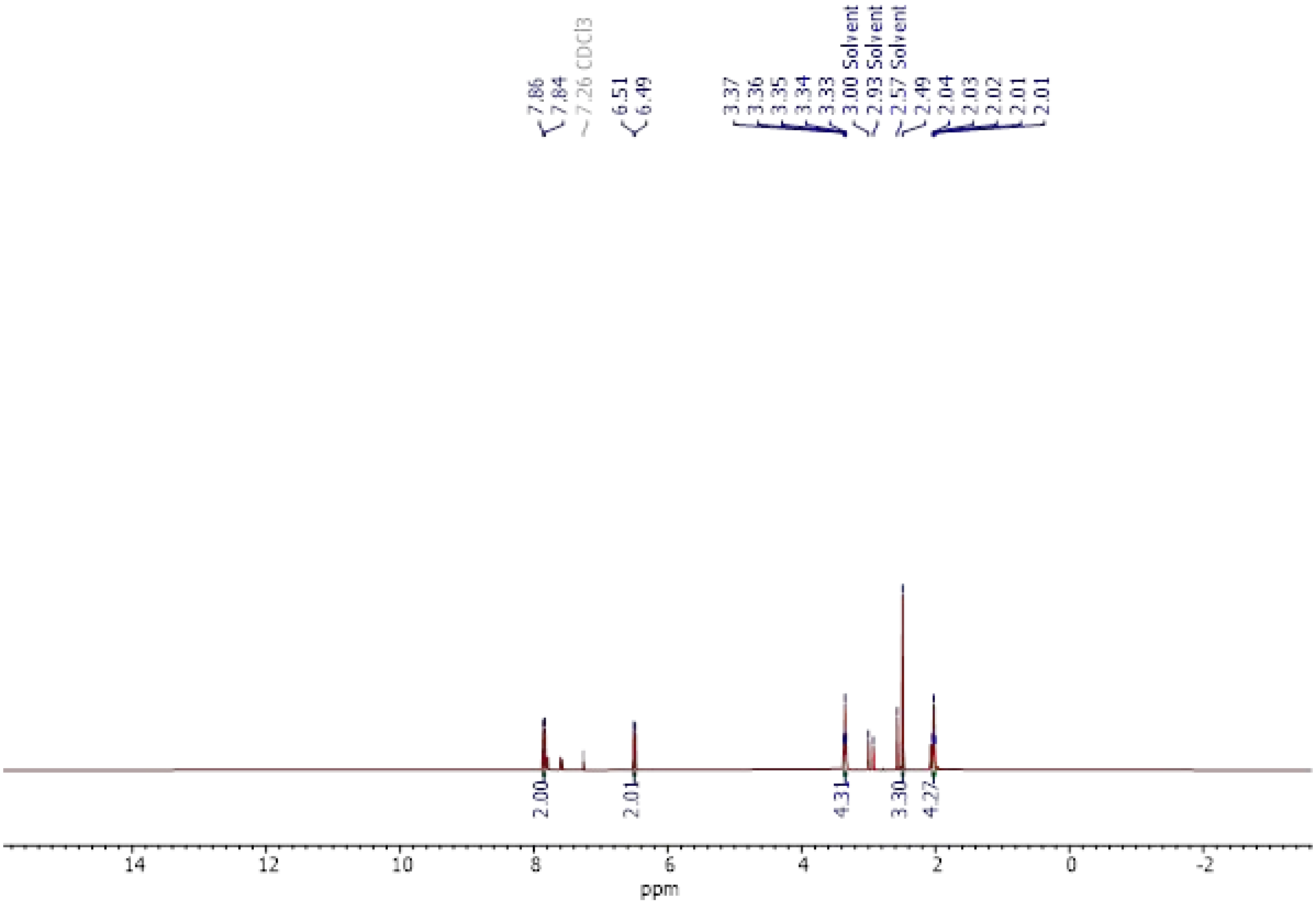

5 [1-(m-tolyl)pyrrolidine]

^{1}H NMR (400 MHz, $CDCl_3$) δ: 7.10 (1H, J = 8 Hz, t), 6.48 (1H, J = 8 Hz, d), 6.38 (2H, J = 5.64 Hz, d), 3.36 – 3.24 (4H, m), 2.30 (3H, s), 1.98 (4H, J = 3.92 Hz, t)

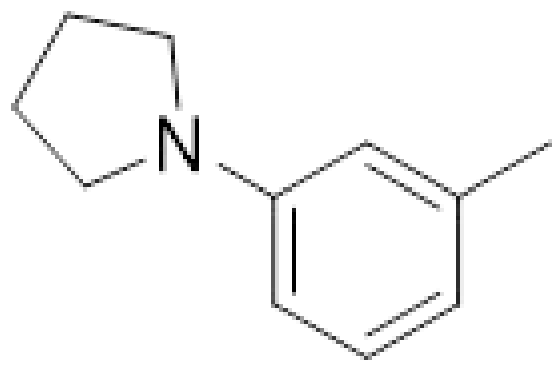

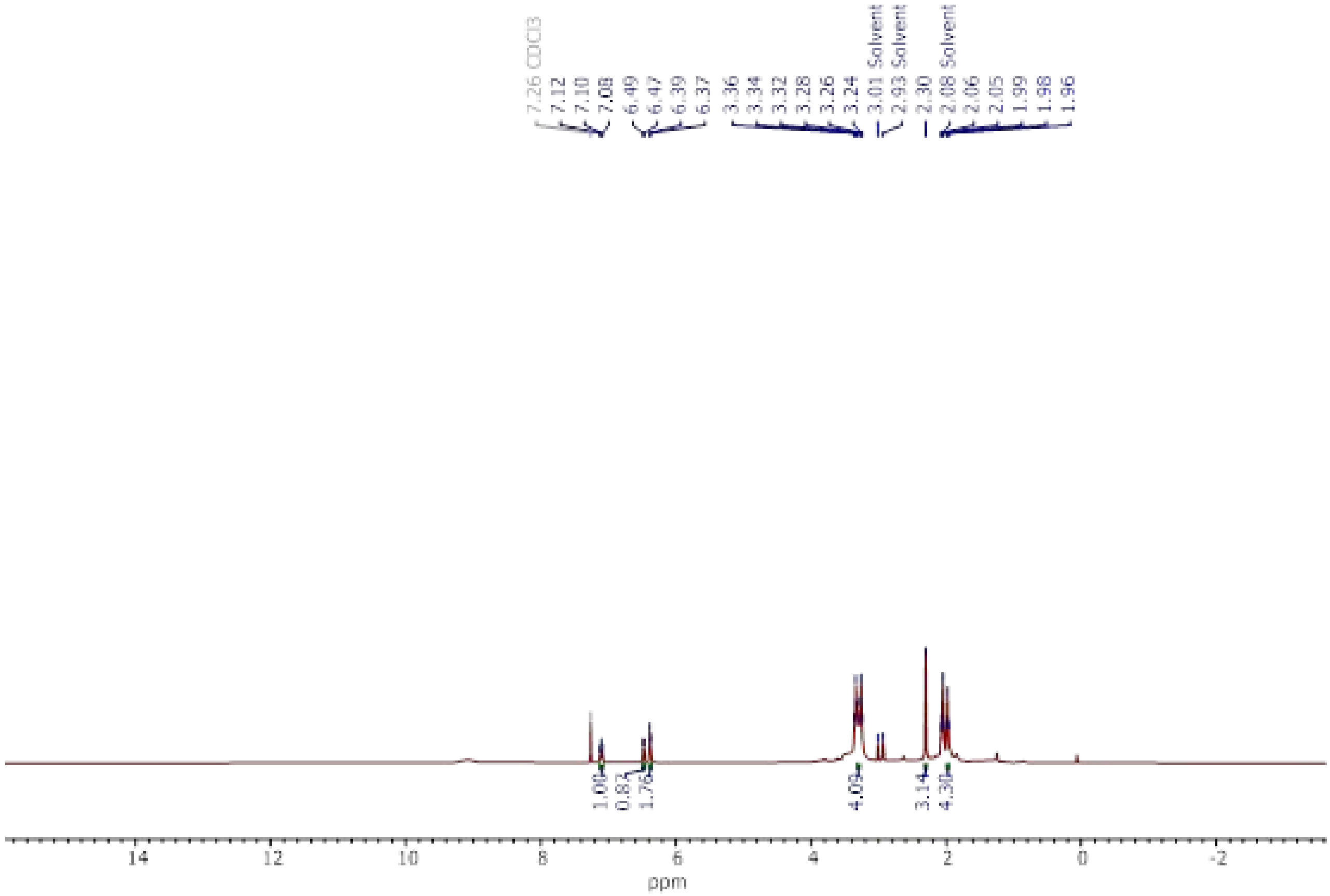

6 [2-(pyrrolidine-1-yl)benzonitrile]

^{1}H NMR (400 MHz, DMSO) δ: 8.04-8.02 (1H, m), 7.98-7.94 (1H, m), 7.32 (1H, J = 8 Hz), 7.25 (1H, J = 8.8 Hz, t), 4.08-4.05 (4H, m), 2.52-2.48 (4H, m)

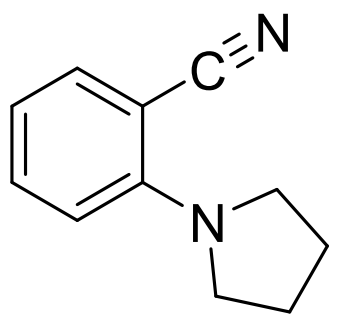

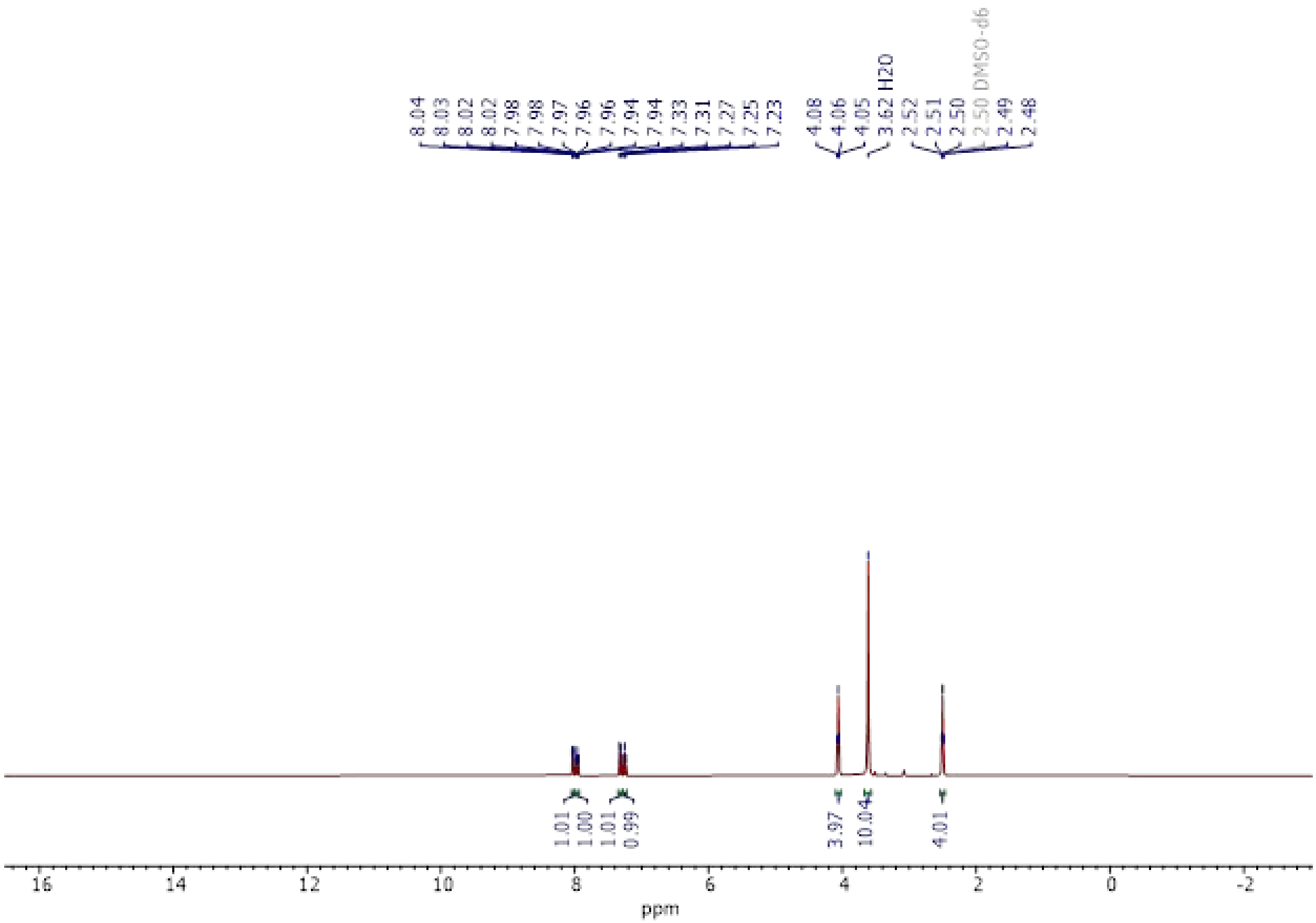

7 [1-(4-bromophenyl)pyrrolidine]

^{1}H NMR (400 MHz, $CDCl_3$) δ: 7.29 (2H, J=8 Hz, d), 6.58 (2H, J=8 Hz, d), 3.41-3.33 (4H, m), 1.95-1.86 (4H, m)

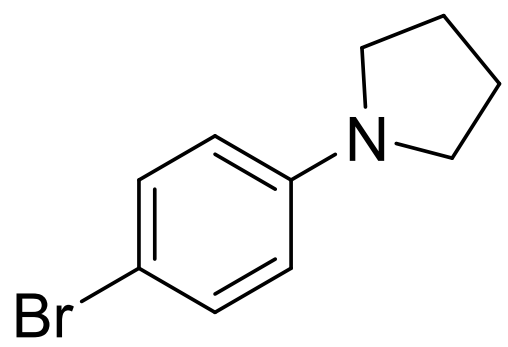

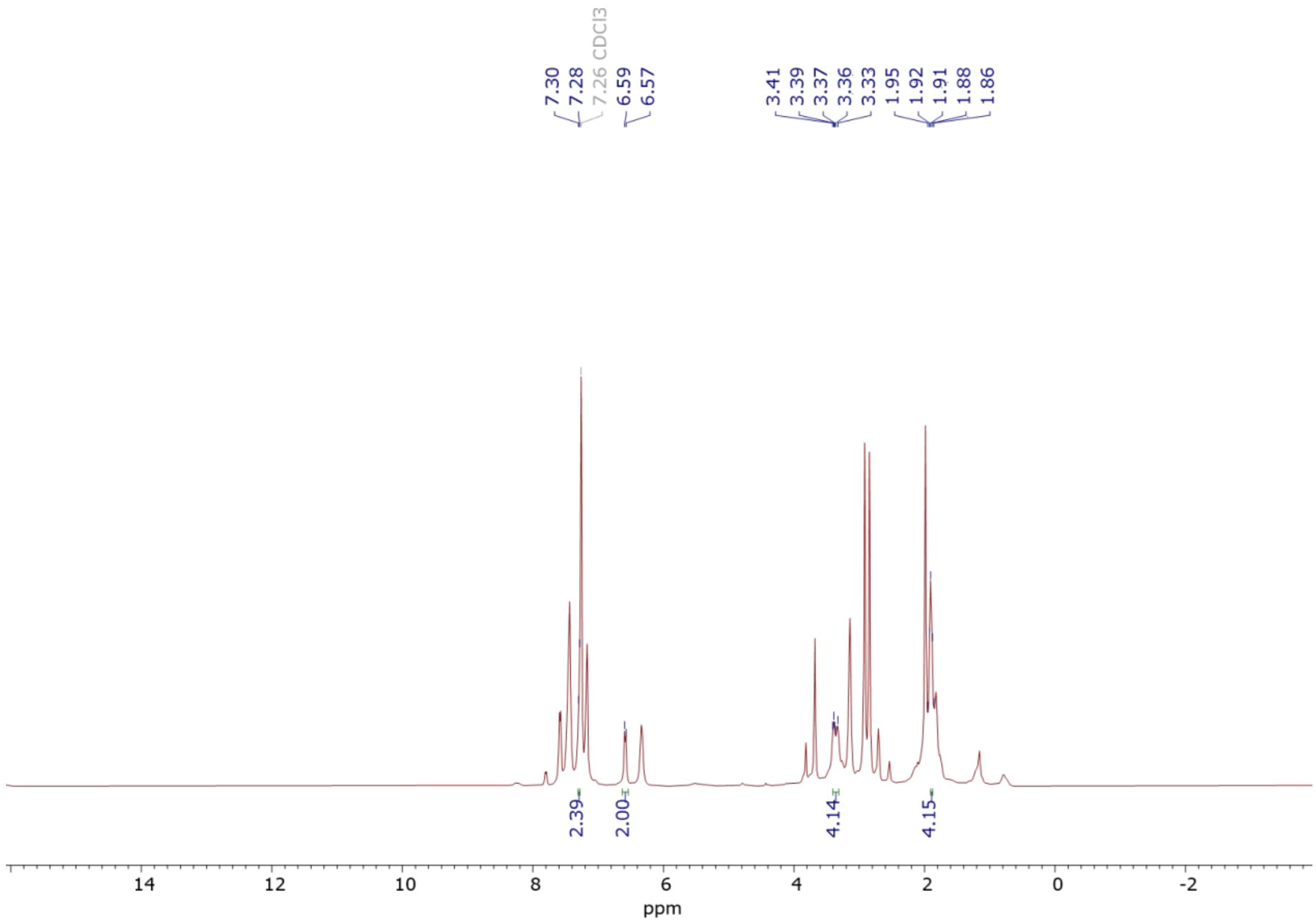

8 [4-(butylamino)benzonitrile]

^{1}H NMR (400 MHz, $CDCl_3$) δ: 7.40 (2H, J = 8.8 Hz, d), 6.54 (2H, J = 8.8 Hz, d), 4.20 (1H, br), 3.14 (2H, J = 7.2 Hz, q), 1.61 (2H, J = 7.2 HZ, quin), 1.42 (2H, J = 7.1 Hz, hex), 0.96 (3H, J = 8 Hz, t)

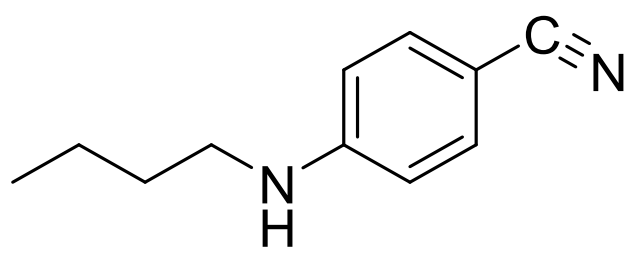

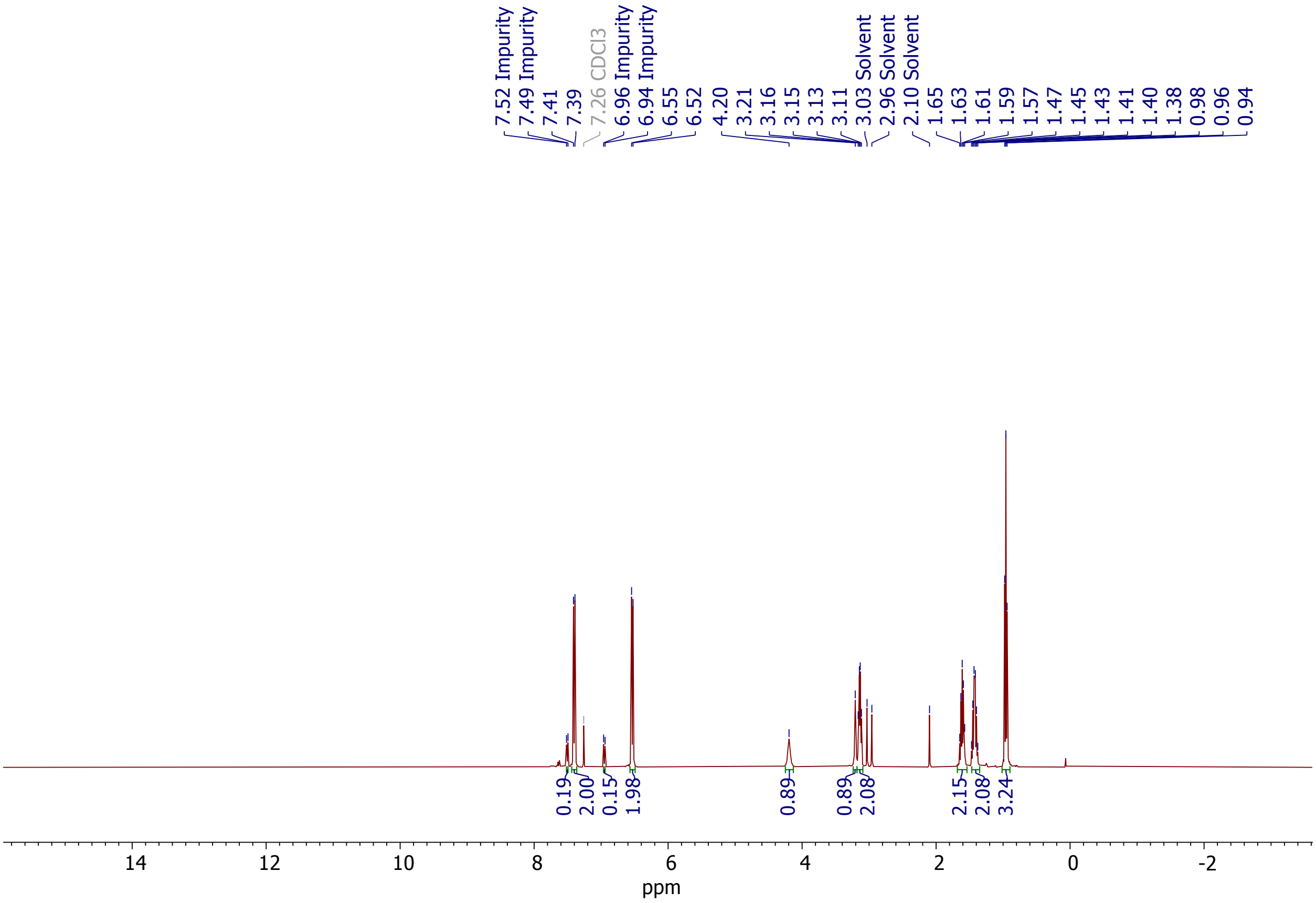

9 [4-(piperidin-1-yl)benzonitrile]

^{1}H NMR (400 MHz, $CDCl_3$) δ: 7.45 (2H, J = 8 Hz, d), 6.83 (2H, J = 8 Hz, d), 3.33-3.31 (4H, m), 1.68-1.65 (6H, m)

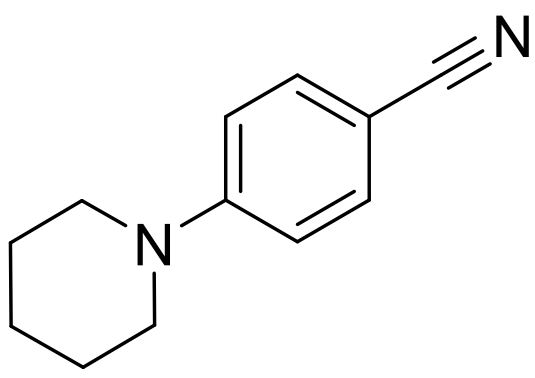

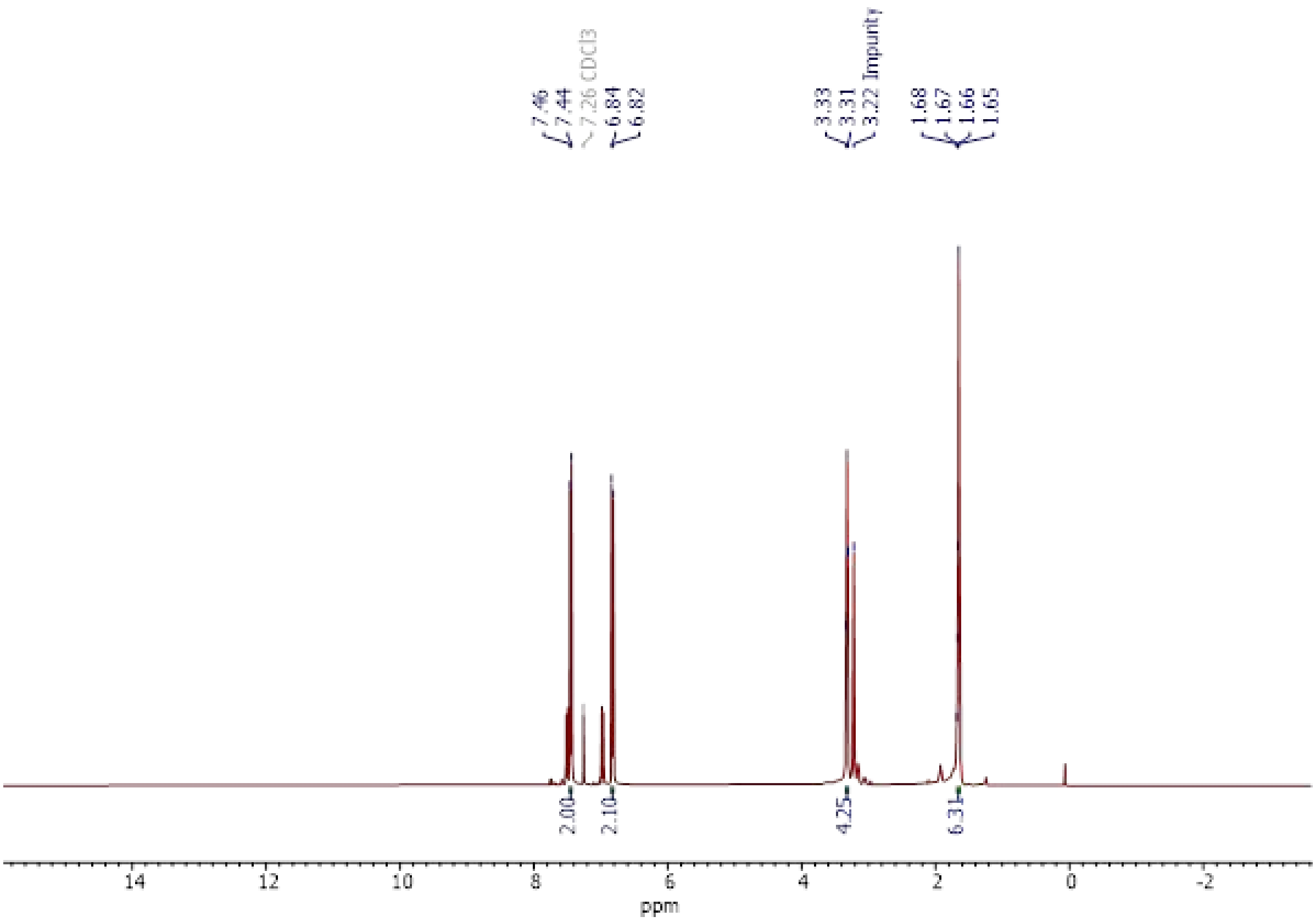

10 [4-morpholinobenzonitrile]

^{1}H NMR (400 MHz, $CDCl_3$) δ: 7.48 (2H, J = 8.8 Hz, d), 6.84 (2H, J = 8 Hz, d), 3.82 (4H, J = 4.2 Hz, t), 3.26 (4H, J = 4.2 Hz, t)

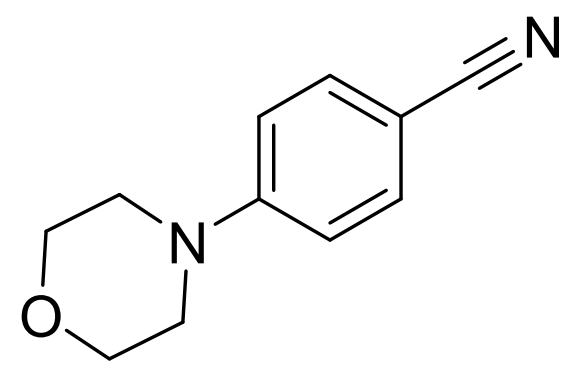

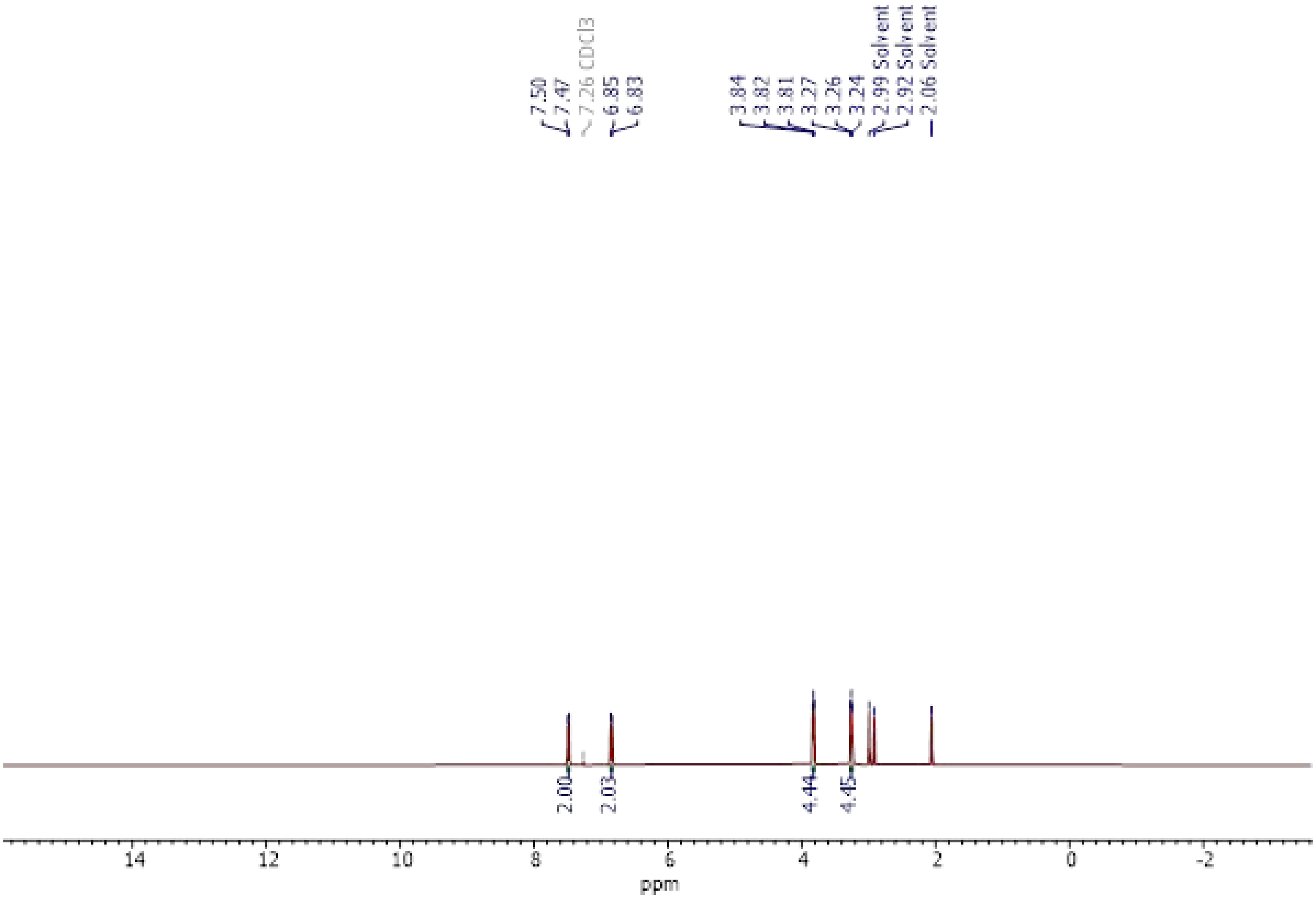

Supplement: Supplementary file 1 [file mg6c00080_si_001.pdf]
